# High-Throughput Screening of Natural Product and Synthetic Molecule Libraries for Antibacterial Drug Discovery

**DOI:** 10.3390/metabo13050625

**Published:** 2023-05-02

**Authors:** Navid J. Ayon

**Affiliations:** Chemistry of Life Processes Institute, Northwestern University, Evanston, IL 60208, USA; nayon@umkc.edu

**Keywords:** automation, antibiotics, high-content screening, natural product screening, chemical library screening, small molecule screening, antimicrobial peptides, bioactivity

## Abstract

Due to the continued emergence of resistance and a lack of new and promising antibiotics, bacterial infection has become a major public threat. High-throughput screening (HTS) allows rapid screening of a large collection of molecules for bioactivity testing and holds promise in antibacterial drug discovery. More than 50% of the antibiotics that are currently available on the market are derived from natural products. However, with the easily discoverable antibiotics being found, finding new antibiotics from natural sources has seen limited success. Finding new natural sources for antibacterial activity testing has also proven to be challenging. In addition to exploring new sources of natural products and synthetic biology, omics technology helped to study the biosynthetic machinery of existing natural sources enabling the construction of unnatural synthesizers of bioactive molecules and the identification of molecular targets of antibacterial agents. On the other hand, newer and smarter strategies have been continuously pursued to screen synthetic molecule libraries for new antibiotics and new druggable targets. Biomimetic conditions are explored to mimic the real infection model to better study the ligand–target interaction to enable the designing of more effective antibacterial drugs. This narrative review describes various traditional and contemporaneous approaches of high-throughput screening of natural products and synthetic molecule libraries for antibacterial drug discovery. It further discusses critical factors for HTS assay design, makes a general recommendation, and discusses possible alternatives to traditional HTS of natural products and synthetic molecule libraries for antibacterial drug discovery.

## 1. Introduction

In the last few decades, bacterial infection has become a critical health concern due to the constantly developing resistance to existing antibiotics and the extremely dried pipeline of new antibacterial agents [1,2,3]. Globally, antibiotic resistance has been reported in bacteria that cause common infections such as urinary tract infections, pneumonia, bloodstream infections, skin infections, etc. [4]. Especially, the human facultative pathogenic bacteria from the ESKAPE group (*Enterococcus faecium*, *Staphylococcus aureus*, *Klebsiella pneumoniae*, *Acinetobacter baumanii*, and *Pseudomonas aeruginosa*, and *Enterobacter species*), which are the causes of a wide range of serious hospital-acquired infections, are also alarmingly becoming resistant to common antibiotics, which is a matter of grave concern [5]. The development of resistance even before the introduction for clinical use as identified against tigecycline demonstrates the ominous situation of bacteria outrunning efforts to prevent them from causing untreatable infections [6]. The continued growth of resistance against existing therapeutics along with the lack of new antibiotics in clinician’s armamentariums are predicted to cost 30 million lives globally by 2050 [7]. Synthetic tailoring of existing chemical scaffolds was successful in tackling resistant pathogens and infections caused by them, and the emergence of multidrug resistance and the severe health risks associated with these multidrug-resistant pathogens warrant urgent mediation [8]. However, efforts undertaken by big biopharmaceutical companies are not picking up because of a lack of success in discovering new antibiotics, low return on investment from antibiotics due to the short course of drug regimens, and very specific treatment opportunities that are restricted to specific bacterial infections. Hence, novel approaches are required urgently to discover new antibacterial agents to fight off infections caused by pathogenic and multidrug-resistant bacteria, and the World Health Organization (WHO) has urged the scientific community to help tackle this situation [4].

With the advent of the state-of-the-art instrumentations and technologies along with the knowledge from the past, novel and smarter approaches are now being sought to explore different routes of drug discovery using strategies not attempted before [9]. Historically, natural products have always been a popular source for bioactivity screening, especially for antibacterial activity [10], and extracts of various plants [11], fungi [12], or bacteria [13] have been tested against different bacterial species. The complexity of the natural product extracts containing a plethora of molecules at varying concentrations with the potential to antagonize or synergize a biological activity, as well as the presence of colored compounds, have been the confounding factors for natural product library (NPL) screening. Additionally, the possibility of rediscovering previously identified bioactive molecules and the time, effort, and resource it takes just to eradicate this possibility leads to a tremendously low return on investment. With all the low-hanging fruits being plucked, it has been a challenge to find new non-toxic bioactive molecules from existing natural sources or to find new natural sources of bioactive molecules. Screening of the synthetic molecule library (SML), on the other hand, gained tremendous attention among the scientific community due to the ability of the rapid screening of thousands of compounds against various microorganisms (whole cell/cellular target) or proteins (molecular target) [14,15,16]. In spite of having a higher hit rate (0.3%) just with polyketide natural products compared to a <0.001% hit rate for synthetic molecule libraries, discovery efforts often favor the screening of synthetic molecule libraries [17]. However, due to a lack of diversity, the SML often fails to identify new bioactive compounds, and this lack of success was also observed in ultra-HTS campaigns undertaken by many big pharmaceutical companies that screen molecules in the range of 10^6^ to 10^7^. Advances in genome science helped to identify and abundantly express unique microbial targets, as well as bioactive molecules; however, there was limited success in producing antibacterial agents [18,19]. Combinatorial chemistry-based HTS has been attempted to increase chemical diversity but has not been very successful in identifying novel antibacterial agents. As a result, antibacterial drug discovery research has come to a quite stagnant stage, reflected by no discoveries of a new class of antibiotics in the last 30 years since the discovery of only a couple of novel scaffolds such as fidaxomicin, retapamulin, and daptomycin [20]. Experts estimate that tens of thousands of species need to be screened to identify new members of known chemical scaffolds [21], which highlights why the collection of new natural sources and creative screening strategies are required for modern day antibacterial drug discovery efforts. Therefore, it is not only of paramount importance to come up with new antibiotics, but to do it at a faster pace.

Singh et al. comprehensively discussed various HTS strategies for microbial natural product libraries until 2011 [22], whereas a comprehensive framework of antibiotic discovery and development can be found in Miethke et al. [23], and the readers are encouraged to consult these excellent reviews. In this narrative review, an extensive effort was made to discuss traditional, as well as new, contemporaneous, and unique strategies to date to screen natural product (diversity) and synthetic molecule (annotated) libraries for antibacterial activities. Various technical aspects to consider for designing HTS assays are discussed with a general recommendation (workflow) to follow some promising alternative and complementary approaches. The aim of this review is to provide an information-rich guide to readers to inform the various ways to conduct high-throughput antibacterial library screening to avail the full scope of these screening platforms and help to decide the best approach that suits the goal of the study. Hence, the focus has been kept on various automated HTS strategies for antibacterial screening in general rather than focusing on any specific natural product reservoir, chemical library source, or any specific bacterial strain. The readers are encouraged to consult other noteworthy review articles in the field of antibacterial drug discovery [9,24,25,26]. A comprehensive discussion on different natural product resources and/or targets is out of the scope of this review paper, and so is the application of other available technologies, such as genomics [27,28] or other omics studies [29,30], which are discussed in great length elsewhere.

## 2. Approaches and Strategies for Antibacterial High-Throughput Screening (HTS) Assays

### 2.1. Cellular and Molecular Target-Based HTS

**Figure 1:**
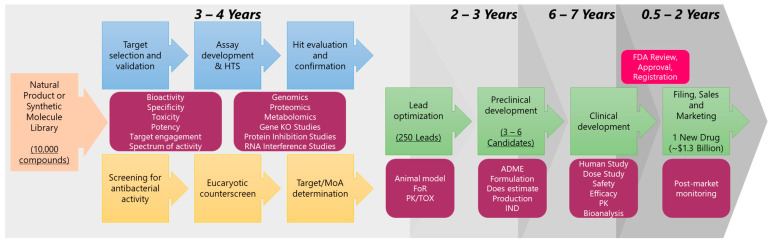
Antibiotic drug discovery and development flowchart. The number of compounds, leads and final drug is arbitrary chosen to depict the success rate in antibacterial drug discovery compared to the time it takes (adapted from the literature [33,34]).

Primarily, there are two approaches for high-throughput screening-based drug discovery assays: (1) the whole cell-based assay, which provides intrinsically active agents for which target identification can be challenging and needs secondary screening to eliminate non-specific cytotoxic compounds [3,18,31], which will be referred as cellular target-based HTS in this review (CT-HTS), and (2) protein, enzyme, or molecular target-based HTS (which will be referred as MT-HTS in this review), which often fails to exhibit bioactivity in vivo due to a number of reasons such as poor permeability, efflux, or the in vivo interaction being dependent on certain conformation, factors, or non-specific bioactivity due to binding to multiple targets [32] that need secondary screening to eliminate pan assay interference molecules (PAINS), such as broad spectrum protease and kinase inhibitors [33]. Following hit identification and hit-to-lead generation, animal studies and clinical trials all are time-consuming processes (Figure 1), and success of which depends on the efficacy, potency, and specificity of the identified molecules (hits), which ultimately depends on the quality and robustness of the HTS assays.

### 2.2. Mechanism Informed Phenotypic HTS Screening (Reporter-Based HTS)

Mechanism-informed phenotypic screening is one of the newer strategies for antibacterial HTS assays, the most common of which is a reporter gene assay that reports the signaling pathways with which the hits are interacting [35,36]. However, a reporter gene assay demonstrates if a molecule is working against a specific pathway for which the molecular target needs to be known, which can have spatial and structural differences from strain to strain [37]. Imaging-based HTS assays that identify antibacterial agents based on their film formation ability [38] or by using a reporter of antibacterial activity, such as adenylate cyclase, that gets released upon cell lysis [39] have been developed and can be used to screen libraries with higher sensitivity and specificity [40] and against biofilm-forming bacteria that can escape antibiotic treatment by hiding in the films. Other reporters, such as molecules interacting with lipids of the cell membrane using a fluorescence anisotropy technique, can be availed to screen antibacterial agents targeting lipid II and interacting proteins (PBP1b, FtsW, and MurJ) [41].

### 2.3. Virulence and Quorum-Sensing Targeting HTS

Screening for virulence factor inhibitors (also known as quorum-sensing inhibitors) is another area where HTS is being explored to help in finding new antibiotics that will either prevent the biosynthesis of autoinducers, degrade them, or compete with them and will bind to the sensors to stop the expression of virulence and quorum-sensing genes. Similarly, antibacterial HTS for molecules to inhibit the ability of bacteria to adhere and colonize the host cells by inhibiting pilus biogenesis, secretion systems, and iron uptake, and genes protecting from the host immune system such as oxidative stress, cationic effectors, etc., are other ways to screen for antibacterial activities. LED209 is a successful example of a quorum-sensing inhibitor that was identified by screening 150,000 molecules using the CT-HTS strategy and demonstrated successful in vivo antibacterial activity against *Salmonella typhimurium* and *Francisella tularensis* [42]. However, it has been addressed that the in vitro HTS assay to find quorum-sensing inhibitors might not always translate into the host [43]. Sonia Escaich discussed various antivirulence molecules identified through HTS of the SML and NPL, as well as virtual screening in greater detail [43], that the readers are encouraged to consult.

### 2.4. Genome Science, Molecular Target Identification, and HTS

Whole cell-based assays utilizing resistant gene-containing strains or strains that lack cell walls have been used to filter out known antibiotics and dereplicate and identify antibacterial agents that target cell wall biosynthesis [44]. Whole genome sequencing has been the method of choice to help identify molecular targets of hits from CT-HTS [45] and the identification of adenosine triphosphate (ATP) synthase as a target of R207910, which demonstrated the utility of this approach among many other studies [46]. The fluorescence polarization technique, on the other hand, demonstrated success in identifying inhibitors of a wide variety of enzymes including GPCR and nuclear receptors, as well as inhibitors of protein–protein interactions [47]. However, they were deemed not suitable for natural product library screening [48]. Genetic manipulation has also been utilized to help finding molecular targets of antibacterial agents, such as sensitive-resistant pair screening (philipimycin), target upregulation or overproduction and gain of the control rescue assay (novobiocin), reduced expression of target or hypersensitive screening (cerulenin), etc. Traditional CT-HTS and MT-HTS assays using either natural products or synthetic molecule libraries have generally been more successful in identifying anti-gram-positive antibacterial agents than anti-gram-negative antibacterial agents with some exceptions [49]. However, HTS using multicopy suppressor containing the genomic library of *E. coli* demonstrated successful identification of antibiotic targets in addition to demonstrating the roles of multidrug efflux pump in reducing the success of antibiotics, a major problem for gram-negative bacteria [50], which in general has a hit rate of 1–2 reduced orders of magnitude more than gram-positive, *S. aureus* [33]. Stone et al. identified two hits by HTS of 19,769 compounds, β-thujaplicin and disulfiram, that inhibit the tetracycline resistance efflux pump in *E. coli* and re-sensitize the bacteria to doxycycline [51].

### 2.5. Combination of HTS Strategies and Multi-Target Hits

Combination strategies utilizing both CT-HTS and MT-HTS approaches are on the rise largely because they complement each other by circumventing their limitations of each other [24] and have been comprehensively discussed by Landeta and Meija-Santana [52]. Combined assays can help eradicate false positives but can be expensive and time-consuming. Pathway-directed HTS is an example of a combined strategy that led to the identification of a new class of d-alanylation inhibitors with the potential to treat Methicillin-resistant *Staphylococcus aureus* (MRSA) infection [53], and 140 non-toxic inhibitors of non-replicating *M. tuberculosis* from a 600,000 compound screening [54]. On the other hand, HTS strategies that screen multiple molecules in combination are in use that identify the actives, which undergo pairwise combination screening along with each other and with inactivity to screen for synergistic or additive agents [55]. Multi-target HTS has been preferred by some scientists, as some diseases have complex etiology and result from the alternation of more than one gene [56]. HTS strategy identifying antibacterial agents that inhibit the interaction of proteins (lipopolysaccharide endotoxin) and cells (CD14) has been reported by Kozuma et al. They discovered three novel compounds, pedopeptin A, B, and Cm from a culture broth of *Pedobacter sp* with a minimum inhibitory concentration (MIC) of 2–4 µg/mL against *E. coli* [57]. HTS assays often surface molecules that exhibit activity against diverse targets such as β-lactamase, malarial protease, dihydrofolate reductase, HIV Tar RNA, thymidylate synthase, kinesin, insulin receptors, tyrosine kinases, farnesyl transferase, gyrase, prions, triosephosphate isomerase, nitric oxide synthase, phosphoinositide 3-kinase, integrase, etc. These multi-target inhibitors such as fullerenes, dyes, and quercetin act on these targets via a mechanism known as aggregate formation, and careful consideration of such an unusual mechanism can help improve screening results [58].

### 2.6. Externally Interceded HTS

Small molecule-treated culture of one bacteria is reported to induce the expression of cryptic bioactive metabolite with activity against a different bacteria, an approach termed bioactivity-coupled high-throughput elicitor screening (HiTES) developed by Moon et al., who tested the supernatant of *S. cebuensis* after treating them with a library of 950 elicitors that were inactive against *E. coli* at the level tested, and identified cebulantin, a cryptic lanthipeptide antibiotic that exhibited antibacterial activity against a panel of gram-negative bacteria [59]. HTS based on low-oxygen recovery, oxygen depletion, or nutrient starvation is being used to screen antibiotics against pathogenic bacteria that can stay dormant and develop phenotypic resistance, such as *M. tuberculosis* [60,61].

## 3. Natural Product Library (NPL) Screening for Antibacterial Drug Discovery

### 3.1. Historical Perspectives and Major Bottlenecks

**Figure 2:**
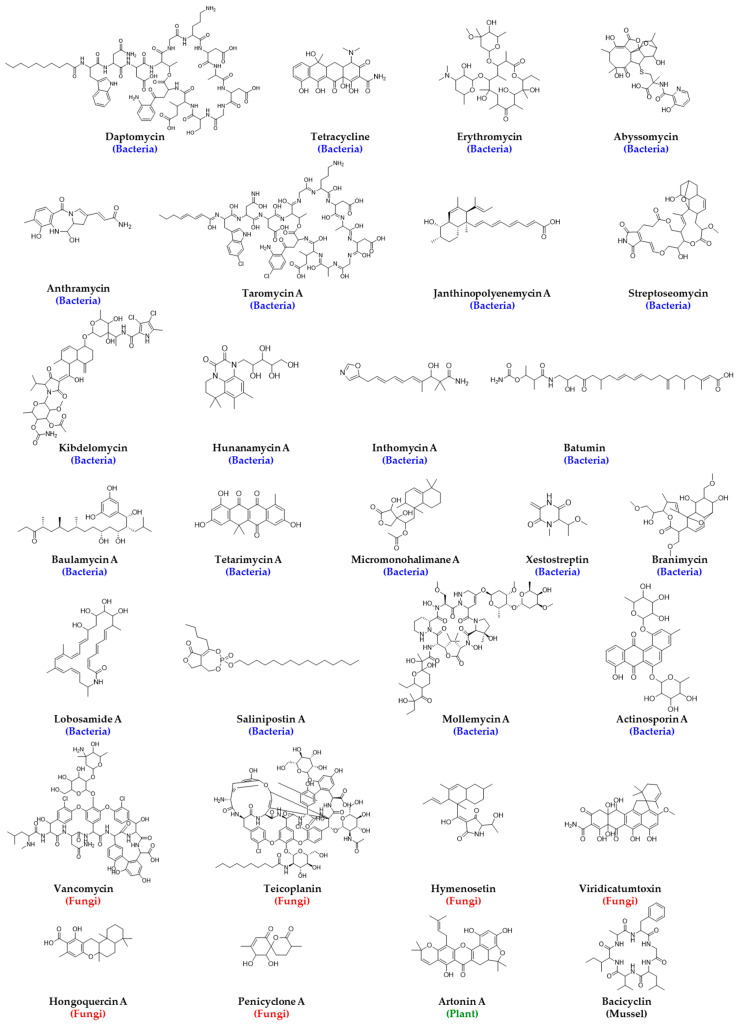
Structures of natural products derived from antimicrobial agents and their natural sources. For molecules with numerous analogs, only the first one in the series, e.g., Taromycin A, is represented structurally.

One of the greatest antibiotic discoveries, penicillin, was identified from fungi, *penicillium notatum,* in 1928, which was followed by the discovery of numerous bioactive chemical scaffolds from microorganisms between 1940 and 1962. Since then, natural products derived from plants, fungi, bacteria, lichens, and endophytes have almost always been screened for antibacterial activities [62], and newer natural sources for drug discovery such as algae, cyanobacteria, and marine invertebrates such as sponges, sea cucumbers, sea urchins, seaweeds, corals, and other marine organisms are being explored for new antibiotics discovery. M. G. Maloney defined the natural products with antibiotic activity as antibiotic-ome [63], and some of the notable members of this new area include daptomycin, tetracycline, erythromycin, vancomycin, teicoplanin, abyssomycin, bacicyclin, anthramycin, taromycin, janthinopolyenemycin, streptoseomycin, hymenosetin, kibdelomycin, hunanamycin, inthomycin, penicyclones, batumin, baulamycin, tetarimycin, artonin, viridicatumtoxin, hongoquercin, micromonohalimane, xestostreptin, branimycin, lobosamide, salinipostin, mollemycin, and actinosporin, (Figure 2) [64]. Rani et al. listed different antibacterial natural products identified until 2021 in their comprehensive review, which the readers are encouraged to consult [65].

From 1982 to 2019, out of 162 antibacterial drugs on the market, 89 were derived from natural products [66]. Although several antibiotics such as β-lactams, aminoglycosides, macrolides, cyclic lipoproteins, etc., have been discovered in bacteria, the pursuit of identifying novel antibacterial chemical scaffolds in bacteria has encountered fewer promising results lately [63,67]. A generous natural source of many antibiotics, actinobacteria, which has been a goldmine for antibiotic drug discovery, ran out of new and functional agents, not because all antibiotics that they produce are already discovered, but probably because the most common and easily obtainable strains are already screened, the molecules they synthesize that are abundant are already identified, and finding new bioactive molecules from the same strains that escaped prior screening or finding uncharted strains have proved to be challenging. Richard H Baltz discussed how actinobacteria should be used to maximize the probability of finding strains that have not been screened already in great detail. Out of 1000 randomly selected actinobacteria, about ten will produce streptomycin and about four will produce tetracycline, which demonstrates the high degree of bioactive compound rediscovery, which is also known as the issue of dereplication [21]. Despite having so much success to derive drugs from natural products, there is a general decline in interest in exploring natural products as drugs by pharmaceutical companies due to various reasons as mentioned in Table 1.

### 3.2. Why Are Natural Products Still Preferred for Drug Discovery despite the Challenges in Screening Them?

Natural products still remain a dark matter to explore in drug discovery research, as they possess excellent specificity and potency compared to synthetic molecules perhaps due to having privileged structures decorated with numerous cyclic systems, multiple chiral centers, non-canonical amino acids, halogenations, and unusual macrocycles [71]. Natural products in general represent a more diverse chemical space representing molecules with reduced hydrophobicity, higher molecular weights, and stereochemical features compared to synthetic drugs and drug-like molecules, with the potential to bind challenging targets due to larger binding surface, polarity, charged sates, and functional groups that are often absent in synthetic molecules [62]. Additionally, due to evolutionary selection and spatial arrangement with the necessary functional groups required for target engagement, even though they are synthesized in different organisms than their targets are housed in, makes them desirable for bioactivity screening [72]. Additionally, naturally derived antibacterial agents in principle should have less tendency to develop resistance, as many of them are produced to defend the organism against unfavorable conditions, including encountering threatening organisms. Hence, natural product drug discovery has remained an active area of research, and the scientific community is pursuing novel ways to collect, grow, and screen new natural sources for drug discovery.

### 3.3. Challenges of Collecting Natural Sources for Screening

**Figure 3:**
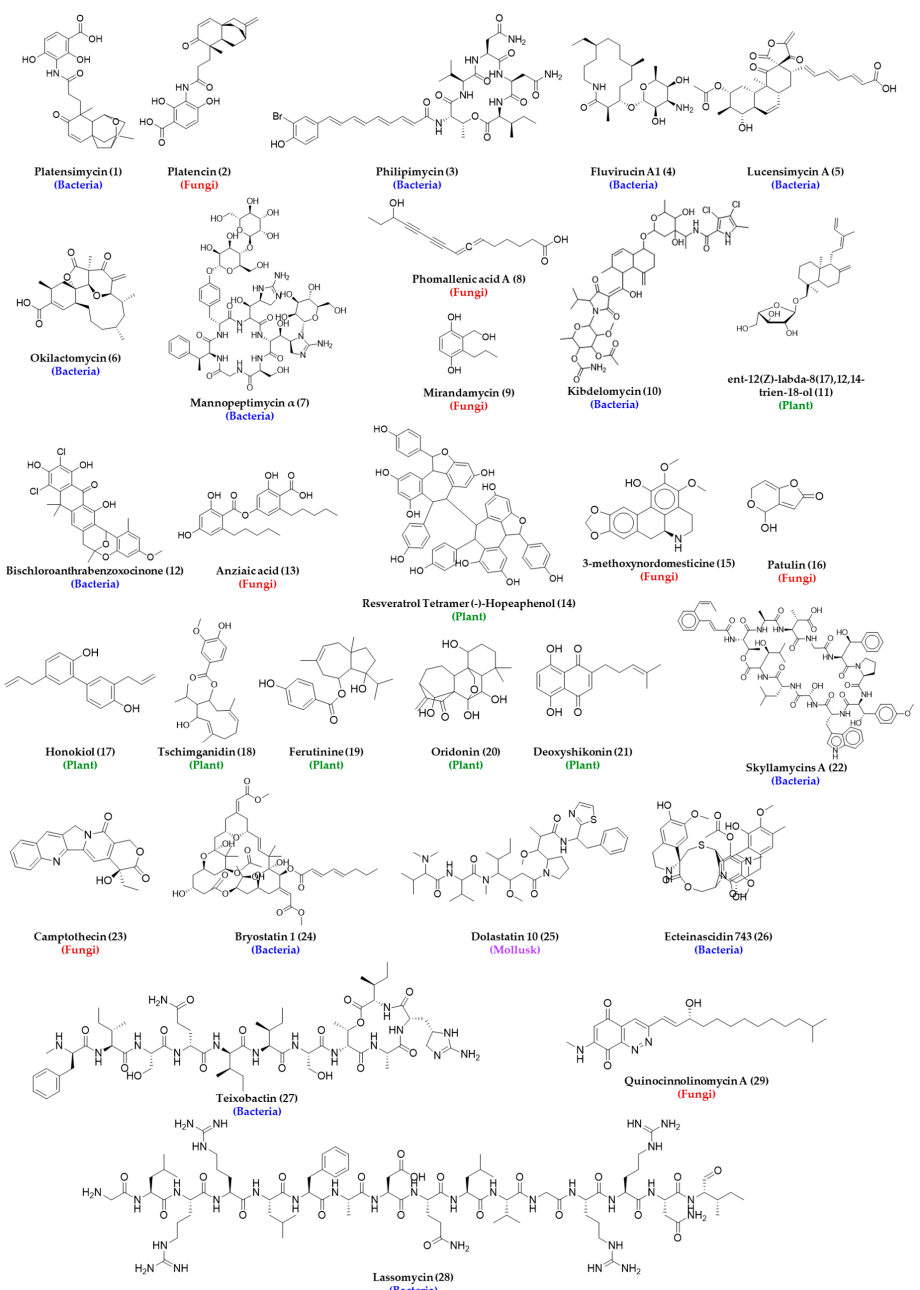
Structures of representative antibacterial agents identified as hits from high-throughput screening of the natural product library. For molecules with numerous analogs, only the first one in the series, e.g., phomallenic acid A, is represented structurally.

The collection of natural bioresources for drug discovery requires sharing, resulting in monetary benefit by the collectors and the host of the bioresource. Collected resources are required to be properly documented and transported, and usage needs to be regulated to prevent the harmful effects of these untested entities. All these make the HTS of natural product extracts quite challenging, which is also reflected by the number of natural product high-throughput screening publications in PubMed remaining below 50 for the period of 1993–2018, whereas high-throughput screening alone had a gradual increase up to above 2000 articles published in 2015 [69]. It has been hypothesized that maximizing biological diversity is crucial to maximizing chemical diversity in natural product libraries, and one way of doing that is through careful phylogenetic selection. As mentioned before, the collection of novel microorganisms from unexplored environments such as deep-sea sediments, desert biomass, hyper-arid deserts, littoral sediments, acidic and alkaline ecosystems, outer space, etc., is extremely challenging and costly. In addition, their culture under laboratory conditions is another challenging task. It has been discussed that less than 1% of all environmental microbes have been cultured under existing routine laboratory conditions, and there may be about 3.7 × 10^30^ microorganisms in the marine environment alone, a great portion of which may synthesize interesting natural products, including potential new drug candidates [73]. Although some of these challenges are nearly impossible to address, scientists are exploring innovative ways to grow new and rare strains or organisms in the laboratory, which is an active research area to date. The addition of nutrients such as pyruvate, cyclic AMP, and homoserine lactones are reported to help with the growth of many microorganisms [74]. A change in growth conditions has demonstrated a shift in metabolome profiles of many microorganisms, such as *Streptomyces griseoviridis,* and can help express cryptic gene clusters leading to the synthesis of new secondary metabolites. The discovery of platensimycin (1), platencin (2), philipimycin (3), fluvirucins (4), lucensimycins (5), and okilactomycin (6) are direct proof of the effect of the growth condition on the production of new antibacterial secondary metabolites [75,76,77]. The structure of the above antibacterial agents and others that are identified from the HTS of the natural product library are listed in Figure 3.

### 3.4. Challenges in Growing Natural Sources under Laboratory Conditions

**Figure 4:**
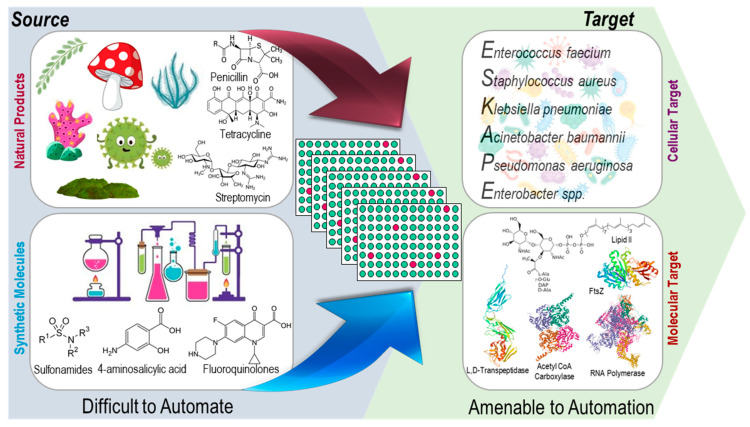
Scope of automation in the preparation of natural products and synthetic molecule libraries for cellular and molecular target-based high-throughput screening. The arrows represent preparation of chemical libraries from natural product and medicinal chemistry efforts in multiwell plate format for screening against cellular or molecular targets for antibacterial drug discovery via HTS.

Due to advancements in the field of genomics, synthetic biology, microbiology, and analytical chemistry, scientists have succeeded in finding strategies to cultivate microorganisms that previously were not possible to culture under laboratory conditions or to induce the biosynthesis of novel secondary metabolites in already explored microorganisms that will not be synthesized under normal conditions. However, finding a growth condition that works for enough natural sources in the HTS setup can be challenging. The manipulation of growth conditions and co-cultures such as plant cell–pathogenic microbes, plant cell–endophytes (or symbionts), and symbiont–pathogenic microbes could ideally lead to the biosynthesis of defensive molecules [78]. Metabolic engineering scientists are exploring genetic and enzymatic engineering to program natural sources to express novel molecules, which is still an area that encounters a lot of challenges. Natural resources that have been reported to provide antibacterial agents include plants, fungi, bacteria, lichens, endophytes, and marine organisms [63,79]. The integration of automation and high throughput for the preparation of natural product extracts is more challenging than that for screening chemical libraries against cellular or molecular targets (Figure 4). Once the extracts from natural sources are obtained as a mixture of molecules, pre-fractioned samples, or pure compounds, the adaptation of HTS for screening against cellular or molecular targets is much more straightforward.

### 3.5. Challenges in Extracting Natural Products

Due to different strains behaving differently under the same growth condition, subcellular localization of desired and undesired metabolites and their membrane transport can be different, which makes the recovery of target products challenging (Figure 3). Certain additives have been reported to help in these situations by drawing out formed products from within the cells such as enzymes, solvents, ionic liquids, or surfactants, which are more amenable for HTS assays [80]. Cell lysis and metabolite extraction using bead beating, lyophilization, freeze/thaw cycling, ultrasonication, microwave-assisted extraction, etc., can be very challenging for incorporating the HTS workflow. Harsher conditions such as boiling solvents, acid-base extraction, steam distillation, etc., were used in the olden days and have replaced by liquid–liquid extraction (in the reactor) or solid-phase extraction (filter plates) for metabolite extraction post-cell lysis, both of which can be automated. Solid-phase extraction has been used for the extraction of microbial secondary metabolites, which are HTS-compatible in 96-well format [81]. Liquid–liquid extraction using methanol/acetonitrile (polar) and methylene chloride, ethyl acetate, tert-butyl methyl ether (TBME) (less polar) with water (to remove protein, carbohydrate, nucleic acids), heptane, and hexane (to remove lipids and fatty acids) can enable the extraction of most drug-like molecules for bioactivity screening [82]. The removal of polyphenols from plants that can interfere with various enzyme assays can be achieved using polyamide or polyvinylpyrrolidone. Resins such as polystyrene-based ion exchange resin have been reported to be used during extraction or as part of the liquid culture of bacteria and fungi to capture metabolites. In general, plants and marine invertebrates can be used as dried powder whereas for bacteria and fungi, liquid or solid culture can directly be used for metabolite extraction. Automated high-throughput extraction procedures used so far include pressurized or accelerated solvent extraction [83,84], ultrasound and microwave-assisted extraction [85,86], counter-current chromatography [87], and supercritical fluid extraction [88], most of which require dedicated core facilities. Tracking bioactive compounds in natural product extracts has benefitted from generations of technological advancements and can be achieved by TLC-based bioautography, HPLC-based assays with online, at-line, and offline detection, as well as affinity-based methods, such as frontal affinity chromatography, pulsed ultrafiltration mass spectrometry, imprinted polymers, and affinity capillary electrophoresis [89]. Information obtained from these experiments such as molecular weight, fragmentation, retention time, and UV profile, along with biological target engagement and database search, tremendously help to identify bioactive molecules in the extracts. We realize how chromatographic separation, nuclear magnetic resonance (NMR), and mass spectrometry (MS)-based identification greatly aids in new drug discovery, as can be seen in the mannopeptimycin **(7)** family of antibiotics, a group of molecules binding to lipid II of the cell wall, designated as mannopeptimycin α−ε with an MIC of 0.25–128 µg/mL against *S. aureus* [90].

The establishment, operation, and maintenance of such infrastructure for HTS require high capital, operational, and maintenance costs that make it challenging to pursue by many academic labs because the investment and involvement of government and/or biotech and pharma companies are needed. The National Program for Natural Products Discovery (NPNPD) of the National Cancer Institute (NCI) successfully demonstrated the entire automated process for high-throughput preparation of a natural product library for bioactivity screening that contains more than 230,000 unique extracts derived from plant, marine, and microbial organisms [69]. The NCI also demonstrated the generation of a prefractionated library consisting of 326,000 fractions for screening as of January 2023 [91]. Other similar-sized prefractionated libraries, such as Nature Bank (202,983 fractions) [92] and the Bioinformatics Institute Singapore prefractionated library (120,000 fractions) [93], are also available for drug discovery campaigns. Rienzo et al. reviewed the preparation of natural product library screening in great length and discussed strategies for high-throughput, small-scale fermentation models, such as commercial BioLector [94] and 15 mL ambr platforms [95], which can help to predict improved strain performance at a scale in between commercial bioreactors and microplates under a well-maintained environment via the regulation of some of the growth parameters including pH, oxygen, temperature, nutrients, airflow, stirring, headspace pressure, aeration, and gas contents [80]. A comparative overview of various automated technologies at different scales for natural product library preparation is listed in Table 2.

### 3.6. Challenges in Preparing a Natural Product Library for HTS

The success of finding novel chemical scaffolds in natural sources greatly depends on maximizing chemical diversity, which largely depends on maximizing biological diversity [74], which can be achieved by a careful selection of organisms from unique, untapped, ecological niches, also known as bioprospecting [96]. Revolutionary technological advancements in automation and robotics have enabled automated streamlined workflow to weigh, transfer, grow, extract, fractionate, and screen natural products for bioactivity [97], which, when combined with bioprospecting, high-throughput mass spectrometry, rapid chromatography, high-performance column chemistry, and genomics, has tremendous potential in discovering bioactive molecules from natural sources. Such automation requires several computer-controlled components including bioreactors, flash chromatography, solid-phase extraction, filtration, and preparative HPLC [98] to obtain the libraries of natural products in multiwell plate format [99,100], which can then be tested using an automated liquid handler followed by spectroscopy-based readouts, and active molecules then can be characterized by NMR, mass spectrometry, and other analytical chemistry tools. NMR spectroscopy and evaporative light scattering detector (ELSD)-based approaches have demonstrated utility in preparing NPL for HTS assays [101]. ELSD-based approaches, in particular, demonstrated success quantifying natural products lacking chromophore and have been coupled with HPLC, MS, and UV for library preparation [102]. Srivastava et al. discussed various strategies for microbial screening to identify chemotherapeutics along with available bioinformatics pipelines [103].

For cellular target-based HTS using NPL, the presence of cytotoxic agents in the extracts can obscure the activity of the bioactive molecules, hence purification followed by a retest, and a cytotoxicity assay is required. On the other hand, for the molecular target-based NPL HTS assay, inhibition of the target can be due to various bioactive compounds acting together or acting against each other [104], and in both cases, further isolation and testing are required, which are time-consuming and expensive with a possibility of identifying previously identified bioactive molecules or a risk of losing bioactive molecules during the isolation process. In spite of so many obstacles, HTS of NPL demonstrated success by the discovery of platensimycin **(1)** as mentioned before, a selective inhibitor of β-ketoacyl-(acyl-carrier-protein) synthase I/II (FabF/B), which was identified by screening 250,000 microbial extracts by Merck [105]. Whether it is a cellular or molecular target-based assay, crude natural product extracts are challenging to screen because they are a complex mixture of many molecules in different amounts without similar reachability to the target with a possibility to show some extent of synergy or antagonism. The pre-fractionated natural product library prepared by various chromatographic techniques demonstrated its potential in identifying bioactivity, which was missed during crude extract screening [106] due to interference from other components in the mixture. For relative simplicity in identifying bioactive natural products and their target engagement, libraries comprising fractions of natural products are often the preferred choice over natural product crude extract libraries, although it is more expensive and time-consuming to prepare and obtain the former.

### 3.7. Available Resources for NPL for Drug Screening Research and Campaigns

Many natural product libraries act as either crude extracts or as fractions and are now available from various federal and private organizations, such as the US National Cancer Institute’s Natural Product Repository comprising >180,000 extracts from >50,000 organisms [69], The Scripps Research Institute [107], Wyeth, which is now Pfizer [108], the Spanish Fundación Medina [109], the Lepetit Research Center [110], the Korean Research Institute of Bioscience and Biotechnology, the Eskitis Institute in Australia [111], and AnaltytiCon Discovery GmbH (20,000 pure natural compounds) [82], among others. Due to years of screening against various pathogenic bacteria and/or molecular targets, these natural product libraries often lead to the rediscovery of known antibacterial agents and are being depleted from new use. Hence, scientists are finding new natural resources or ways to exploit biochemical machinery of the existing natural resources such as genetic manipulation, feeding experimental precursors, varying growth conditions, coculture, etc., which can help to express mutated or cryptic biosynthetic gene clusters leading to new bioactive metabolites and hold potential for further testing [112,113]. Libraries consisting of chemically synthesized molecules inspired from natural products, such as libraries based on core scaffolds, with general structural characteristics of natural products and specific structural motifs from natural product classes, were discussed by Derek Tan with numerous examples of successful identification of bioactive molecules against various biological targets across various disease areas [114].

### 3.8. Examples of Successful HTS of NPL for Antibacterial Drug Discovery

Despite the challenges, there are plenty of examples where natural product crude extract libraries were successful in providing antibacterial agents, as can be seen in the following discussions, Figure 3 and Table 3. Nybond et al. reported a bioluminescent whole cell reporter gene assay that could identify antibacterial substances from the plant natural product library within 2–4 h at a concentration of 50–100 µg/mL [115]. Young et al. reported the identification of FavH/FabF inhibitors and phomallenic acid A (8), B, and C using a parallel agar plate-based HTS using the control and fabF mutant strain by screening over 250,000 natural product extracts (plants and fungi) with a broad spectrum of activity against *S. aureus*, *E. coli*, *H. influenzae,* and *B. subtilis* [116]. Yemele-Leki et al. developed a simple, robust, and inexpensive colorimetric HTS assay, screened 39,000 crude extracts derived from different organisms, and identified 49 extracts with antibacterial activities leading to the identification mirandamycin (9), which is active against *E. coli*, *P. aeruginosa*, *Vibrio cholera*, MRSA, and *Mycobacterium tuberculosis* [117]. This study is one of many examples of how automation can be incorporated after crude extracts were obtained manually from natural sources. Phillips et al. discovered a novel bacterial type II topoisomerase inhibitor, kibdelomycin (10) from the *Kibdelosporangium* strain using antisense-induced strain sensitivity profiling that has broad activity against various bacteria including *S. aureus*, MRSA, *S. pneumoniae*, *E. faecalis*, and *H. influenzae* with an MIC range of 0.5–2 µg/mL with a very low frequency of resistance [118]. Arai et al. identified two antibacterial natural products, ent-12(Z)-labda-8(17),12,14-trien-18-ol (11) and ent-12(E)-labda-8(17),12,14-trien18-ol, from *Limocharis flava* extract using nuclear receptor protein (hVDR) immobilized on magnetic beads followed by reverse phase chromatography [119]. Table 3 lists additional successful NPL screening assays for antibacterial drug discovery.

### 3.9. Antibacterial Biofilm Inhibitory Compounds from HTS of NPL

NPL screening also led to the identification of biofilm inhibitory compounds, such as patulin (16), that can inhibit biofilm formation and detach preformed biofilm by *Salmonella Enteritidis* [109] and honokiol (17), tschimganidin (18), ferutinin (19), oridonin (20), and deoxyshikonin (21) that can inhibit biofilm formation by *S. aureus*, among which ferutinin demonstrated reduced biofilm formation on a catheter in the presence of neutrophils [126]. Harrison et al. developed a miniaturized biofilm model for HTS for antibacterial activity using biofilms grown on PEG lids that can be assessed by measurement of optical density or viable cell counting [127]. Navarro et al. also reported an HTS assay to identify biofilm inhibitors and inducers of biofilm detachment using imaging technology with a marine microbial natural product library against *P. aeruginosa* and identified three cyclic depsipeptides: skyllamycins A (22), B, and C [38].

### 3.10. Antibacterial Agents from HTS of Unconventional Natural Sources

Enhanced diversity by including rare and highly underexplored sources such as terrestrial myxobacteria, marine microorganisms, or any other organisms that exist in extreme environments, e.g., at the deep sea level, in thermal vents, or in salty lakes, which due to extreme pressure, temperature, or pH lead to the synthesis of biomolecules that would otherwise not grow and are uncommon to be produced by organisms found in easily accessible sources such as in soil, woods, lakes, etc., helped to find novel molecules with antibacterial activity. Organisms collected from unconventional sources and genetically engineered libraries have been proven to broaden phylogenetic diversity. The utility of including these rare and/or uncommon natural sources can be realized from the discovery of haloduracin, a two-component lantibiotic from *Bacillus halodurans* [128], topotecan, irinotecan, and camptothecin (23) from terrestrial sources and bryostatin 1 (24), dolastatin 10 (25), and ecteinascidin 743 (26) from marine sources, which are now in clinical trials [129]. Gabriela Simões screened organic and aqueous extracts of 1135 marine microorganisms obtained from 840 to 2300 m below sea level against *Enterococcus* sp. (VanA+), *Klebsiella oxytoca*, *Salmonella enteritidis*, *Salmonella typhimurium,* and *Shigella* sp. and found bioactivities in the extract of a deep sea mussel, *Bathymodiolus azoricus,* and a group of crustaceans, *Microcaris* sp. [130]. In addition to the challenges involved in their collection due to accessibility challenges, they also require dedicated infrastructure and skilled personnel, and from hit to lead generation, these types of molecules can greatly benefit from synthetic and medicinal chemistry approaches. The story of laboratory synthesis of the natural product discodermolide, an antitumor polyketide from the Caribbean sponge, *Discordermia dissoluta,* in multigram quantities is very inspiring and demonstrates the successful synthetic scale-up of a bioactive natural product for clinical studies [131].

### 3.11. Metagenomics and Metabologenomics Aided NPL HTS for Antibacterial Drug Discovery

**Figure 5:**
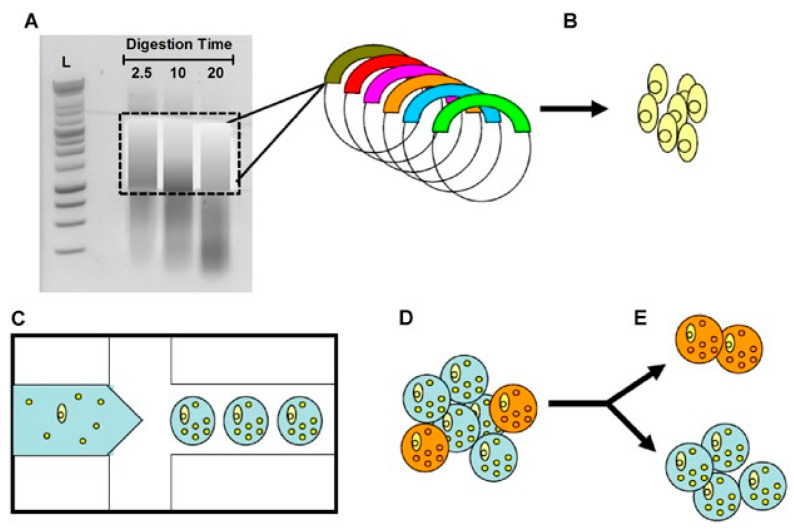
A schematic of an ultra-high-throughput metagenomic screen for antibiotic drug discovery. (**A**) Environmental DNA was subjected to a limited DNaseI digestion, and the size was selected for fragments between 1000 and 5000 bp. (**B**) Metagenomic DNA was cloned into an expression vector by blunt end ligation and then transformed into *E. coli* bacterial hosts. (**C**) A microfluidic device was used to generate agarose-in-oil micro-emulsions in which individual recombinant *E. coli* were co-encapsulated with live *S. aureus* bacterial targets (small yellow spheres). (**D**) Gel microdroplets (GMDs) in which the recombinant clone secretes an enzyme that is lytic toward *S. aureus* and were labeled with the SyTox Orange viability probe, which selectively stains only dead bacteria (orange circles). (**E**) Mixed GMD populations were sorted by FACS to isolate *E. coli*-secreting bactericidal natural products. Genes from the sorted GMDs can be cloned, recombined, or rescreened iteratively to facilitate antibiotic drug discovery [133]. (Reprinted with permission).

A genetically engineered library of microorganisms allows for the expression of biosynthetic genes, such as polyketide synthase (PKS) and biosynthetic gene clusters (BGCs), and such an approach has a promising scope to discover novel molecules [132]. Scanlon et al. reported an ultra HTS platform where they screened 5 million clones of recombinant microorganisms per day against MRSA utilizing microfluidic technology and fluorescence-activated cell sorting (FACS); and identified antibiotic-producing yeast (Figure 5) [133] using metagenomics [134]. FACS-based HTS also demonstrated rapid identification of antibacterial targets by the selective isolation of mutant strains followed by DNA sequencing [135].

Our group has pioneered integrating genomics with mass spectrometry-based metabolomics, also known as metabologenomics, and we identified a new bioactive natural product, tambromycin, and its biosynthetic gene clusters in 11 different actinomycetes that were reported to have anticancer activity; however, no antibacterial activity against different bacterial strains was tested [136]. The Metabologenomics approach has already been extended to other bacterial [137] and fungal species [12] and is used to compare the chemical diversity and biosynthetic landscapes between them [138]. Metabologenomics, along with heterologous expression and biosynthetic pathway manipulation enabled through CRISPR/CAS9, are being used to discover new bioactive natural products and to create a natural product library [139,140].

### 3.12. Microfabricated Chip-Based HTS of NPL for Antibacterial Drug Discovery from Uncultivable Organisms

**Figure 6:**
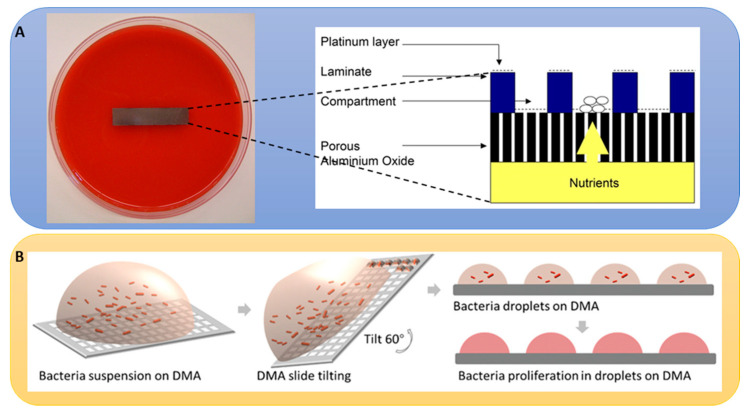
Culture chip use and manufacturing. (**A**) A photograph of a platinum-coated chip placed on a sheep’s blood agar in a standard Petri dish. A cross-sectional diagram of a small part of the chip, illustrating microbial growth in the central compartment (7–150 µm wide) and the supply of nutrients from beneath the 60 µm thickness of the aluminum oxide and the 10 µm high walls. (**B**) A scheme showing the seeing and growth of P. aeruginosa PAO1 GFP on droplet microarray (DMA) slides [146,147]. (Reprinted with permission, Figure 7A—Copyright (2007) National Academy of Sciences, USA).

Ultrafast microfabricated chip-based screening demonstrated the rapid identification of the antibiotic sensitivity of bacteria in less than 20 min, as this technique is sensitive to cell growth and does not require the cells to divide, which is suitable for suspended, adherent, and cultured cells in glass coverslips [141]. One of the most interesting antibacterial agent discoveries from a natural source was teixobactin (27), a cell wall biosynthesis inhibitor that binds to lipid II and lipid III, reported by Ling et al., which was identified when an uncultured bacterium was cultured in its natural soil environment using a multichannel device called isolation chip (iChip) from the extracts of 10,000 isolates against *S. aureus* [142]. The iChip has a central plate with hundreds of through holes to house microbes from the environment that are separated by a semi-permeable membrane and enables the growth and isolation of 384 microorganisms in parallel in the microdiffusion chamber, which was previously uncultivable in a variety of environments and can recover 50% growth compared to 1% that grow under laboratory conditions [143]. This technology can greatly aid in culturing and exploring microorganisms that still remain understudied and can lead to the discovery of new bioactive molecules, as can be seen by the discovery of lassomycin (28), an ATP-dependent protease complex inhibitor [144]. The disc volatization method using Petri dishes demonstrated the successful identification of antibacterial and antifungal natural products [145]. Miniaturized HTS that uses a micro Petri dish contains a million-well growth chip and is reported to isolate β-galactosidase-producing microcolonies from 12,000 clones and phosphate-metabolizing strains from 207,000 microcolonies (Figure 6A) [146].

The microbial fuel cell-based technique utilizing paper and a 48-well system demonstrated the potential of characterizing living cells by recording the ability of microbes to generate electricity [148]. More recently, droplet microarray (DMA) technology has been developed that is compatible with a colorimetric readout of nanoliter droplet-containing bacteria (Figure 6B) [147]. After validating that the number of bacterial cells in a 1 mm hydrophilic droplet was comparable to that from a 96-well plate, Lei et al. screened antibiotics in nanoliter scale against nanoliter bacterial suspension containing 1.8 × 10^9^ ± 0.9 × 10^9^ CFU mL^−1^ using I-DOT and CellScreenChip (CSC). Yüce and Morlock developed a high-performance thin layer chromatography-based high-throughput antibacterial assay that enabled the synthesis, identification, and screening of products from 60 organic reactions at the nano-mole level in parallel with the successful identification of antibacterial agents against *B. subtilis* and *Aliivibrio fischeri* [149]. These miniaturized approaches are extremely smart ways to screen antibacterial activities and hold potential in the antibacterial drug discovery field using a very small material (nano to picoliter) with a single bacterial cell that can screen one million compounds in a day [150].

HTS of NPL has also demonstrated success in discovering hits against unusual targets, as reported by Brandi et al., who developed a cell-free system to screen for inhibitors that target the bacterial translation apparatus and identified two hydrophilic oligopeptides from 25,000 natural product extracts, namely GE-81112 and GE-82832, which bind to different regions of 30S ribosomal subunits and inhibit in vivo protein synthesis [151,152,153]. This indicates the promise of the translation apparatus as a potential target for HTS campaigns.

### 3.13. Coculture-Based HTS of NPL for Antibacterial Drug Discovery

**Figure 7:**
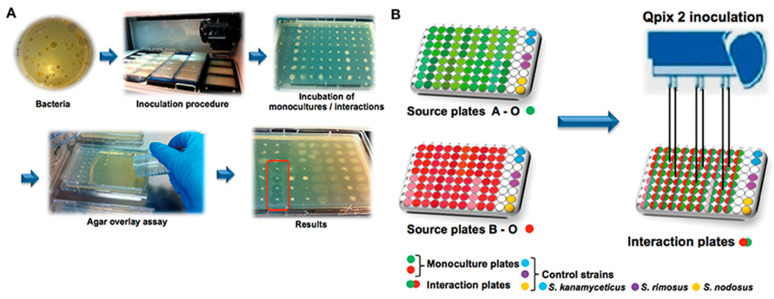
Workflow of the high-throughput interaction assay. (**A**) An overview of the antimicrobial screening: bacteria were inoculated with a Genetix Qpix 2 colony-picking robot either in monoculture or one-to-one interactions on OmniTray™ plates. For the detection of antimicrobial activity, an agar overlay assay with two target organisms was performed on the fourth day of incubation. Antimicrobial activity was determined on the 5th day after overnight incubation at 37 °C by screening for visible zones of inhibition (ZOI) in the upper agar layer. (**B**) An overview of the 96-well plate design and the inoculation procedure using the Genetix Qpix2 colony-picking robot [154]. (Reprinted with permission).

Among other less expensive approaches, coculturing bacterial strains for the potential expression of silent genes and inducing resistance to specifically induce biosynthesis of cryptic molecules have been pursued [20,154]. Tyc et al. reported a high-throughput screening of 146 phylogenetically different soil bacteria in coculture, and found non-induced production, induced production, and suppression using an agar-based method in the 96-well plate format, and studied 2798 unique interactions against *E. coli* and *S. aureus (*Figure 7) [154].

**Figure 8:**
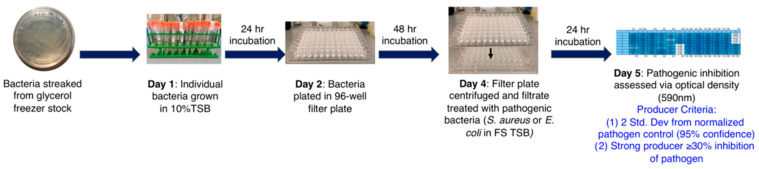
Workflow of the monoculture liquid high-throughput screening method. The five-day process starts after the colonies are quadrant-streaked from agar plates and allowed to grow for 36–48 h. Hits are determined as any inhibition that is two standard deviations from the pathogen in tryptic soy broth (TSB) media. Strong producers are categorized as those hits that have ≥30% inhibition [155]. (Reprinted with permission).

An agar-based HTS of a mono or coculture of bacteria, however, can be time-consuming, more prone to errors, and require manual assessment of the zone of inhibition; however, careful execution can help identify antibiotics-producing microorganisms. Adopting this, Murray et al. screened a bacterial library comprising 260 different strains of mono and coculture using liquid broth against *E. coli* and *S. aureus* and reported antibiotics production from *Pseudomonas*, *Serratia*, *Bacillus*, *Herbaspirillum,* and *Kluyvera* species by rRNA sequencing (Figure 8) [155]. This approach can be easily integrated with high-throughput automation by using liquid handling robotics and can be easily extended to a larger library.

### 3.14. Integrated Platforms for HTS of NPL for Antibacterial Drug Discovery

In order to help with the dereplication and identification of the molecular target, the two most critical aspects of HTS assays, scientists are developing different platforms based on activity, imaging, and metabolomics and leveraging the power of technological and informatic advancements. Mass spectrometry, liquid chromatography, and nuclear magnetic resonance spectroscopy greatly facilitated HTS of natural products including diversity evaluation, dereplication, structure elucidation, fractionation, isolation, and biological activity evaluation [156,157]. Combining the power of this technology helped emerging integrated imaging and informatic and visual platforms, such as Compound Activity Mapping and BioMap, which can help predict the identity of the bioactive molecules and their mode of action from any complex natural product extract library that can bring an early breakthrough in the drug discovery process [158]. These approaches enabled the discovery of naphthoquinone-based antibacterial agents and quinocinnolinomycins (29) that possess unique carbon skeletons [159]. Zhang’s group developed an HTS assay that identified four natural products: xanthohumol, deoxyshikonin, isorhapontigenin, and calycosin from the screening of 1261 natural products, which did not exhibit direct antibacterial activity but significantly augmented the antibacterial activity of porcine macrophages against both gram-positive and gram-negative bacteria [160,161]. Urban et al. reported five *Bacillus subtilis* promoters for the major biosynthetic pathways of bacteria: yorB promoter (DNA biosynthesis), yvgS promoter (RNA biosynthesis), yheI promoter (protein biosynthesis), ypuA promoter (cell wall biosynthesis), and fabHB promoter (fatty acid biosynthesis), and screened 14,000 natural products and identified 6% of the library as active against *B. subtilis* with an MIC < 25 µg/mL [162]. All these notable studies exhibit how natural products can be harnessed smartly to explore new antibacterial agents by widening the molecular landscape under investigation, which indicates that natural products are still a potential source of new antibiotics [163,164,165]. One needs to consider the challenges, complexity, and costs associated with adopting HTS of a natural product library for antibacterial drug discovery, and target identification and advanced analytical chemistry tools, along with bioinformatics, can play a crucial role in early dereplication that can save time and money.

## 4. Synthetic Molecule Library (SML) Screening for Antibacterial Drug Discovery

### 4.1. Historical Perspective and Available Resources

**Figure 9:**
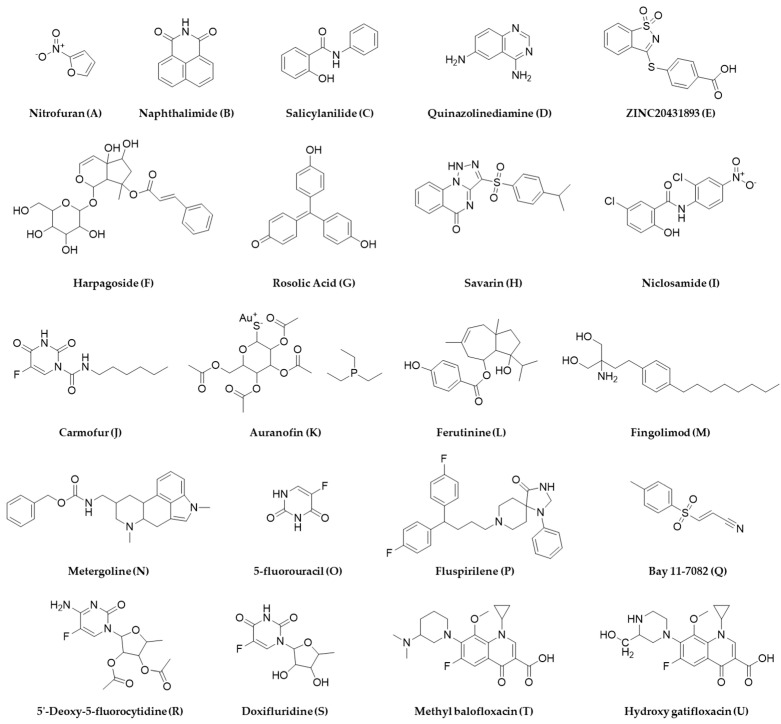
Structures of representative antimicrobial agents identified as hits from high-throughput screening of the synthetic molecule library.

Due to increased time, effort, and cost to completely characterize a novel antibacterial agent from natural sources, challenges with automation, target identification, and dereplication, high-throughput screening of a synthetic molecule library is often preferred, which allows for rapid screening of a large chemical space against a target [166]. Synthetic molecule libraries are much more affordable and can be screened with little or no modification after procurement. HTS of the SML is now extensively used in pharmaceutical industries to discover new drugs or repurpose old drugs for new indications [167]. Due to its popularity in drug discovery, synthetic molecule libraries of different sizes are now commercially available from different vendors such as Thermo Fisher Scientific, Shelleckchem, Apexbio, MedChemExpress, TargetMol, Life Chemicals, Chem Faces, and Samdi Tech, among others, not to mention from the National Institute of Health Chemical Genomics Center, which plays a major role in HTS globally, extending from procurement, quality control, and storage, to the distribution of compounds for research and development purposes [168]. The biggest resource for the synthetic molecule library for antimicrobial drug discovery research is the Community for Open Antimicrobial Drug Discovery (CO-ADD), which allows access to millions of compounds made by organic chemists for whom making new molecules are more important than the molecules to have drug-like properties [169]. The CO-ADD enables access to the 29 million compounds with antibacterial-like properties [170], is an open-access antimicrobial screening program which was launched in 2015, and is funded by the Wellcome Trust and the University of Queensland, where synthetic compounds are being screened using HTS and is free of cost with no incumbent on intellectual property. Similar other programs such as GSK’s Open Lab Foundation, Eli Lilly’s Open Lab, the US NIH Molecular Library Program, the Tropical Disease Initiative, Collaborative Drug Discovery, and the EU-OPENSCREEN are also available for aiding and assisting in drug discovery initiatives; however, the CO-ADD only focuses on antimicrobial drug screening, which makes it unique and noteworthy in the premise of this review. The structures of several antibacterial agents identified from HTS of the synthetic molecule library that are discussed in this section are listed in Figure 9.

### 4.2. Cellular Target-Based HTS (CT-HTS) of the Synthetic Molecule Library (SML)

More than a decade ago, La Fuente et al. reported a turbidimetric HTS assay by screening a diverse collection of 150,000 small molecules and reported antibacterial activity of nitrofuran (A), naphthalimide (B), salicylanilide (C), bipyridinium, and quinoazolinediamine (D) chemical classes, which were more potent against *E. coli* (ATCC 25922) than *P. aeruginosa* (ATCC 15692) at the concentration tested (12.5 µM) [171]. With a similar approach, Malapaka et al. reported the identification of 70 known antibacterial compounds against *Salmonella typhimurium* and validated their approach of detecting compounds with antibacterial and antimotility properties with a standard absorbance microplate reader [172]. Wang et al. reported a comparative HTS assay by screening >150,000 compounds against gram-positive *S. aureus* (ATCC 12608), gram-negative *A. baumannii* (ATCC 19606), and *E. coli* BL 21 (DE3) and identified several novel scaffolds exhibiting antibacterial activities [173]. The National Institute of Allergy and Infectious Diseases (NIAID) and the Tuberculosis Antimicrobial Acquisition and Coordinating Facility (TAACF) conducted a comprehensive HTS of similar magnitude using a 215,110 compound library followed by a 100,997 compound library against *M. tuberculosis* strain H37Rv through the Molecular Libraries Screening Center Network and identified various chemical scaffolds [174,175]. A similar but independent study led by Kotapalli et al. also identified hits that overlapped with some of the identified chemical scaffolds from the TAACF study against *Mycobacterium smegmatis*, *Mycobacterium bovis* (BCG), and *M. tuberculosis* (H37Rv) [176]. Sharma et al. identified fifteen chemical scaffolds and seven compounds with an MIC of less than 1 µg/mL by screening 30,000 compounds against a non-replicating streptomycin-starved 18b (SS18b) mutant strain of *M. tuberculosis*, all of which were non-toxic in the HepG2 cell line [60]. Morgan et al. screened the NIH Molecular Libraries—Small Molecule Repository comprising 292,740 compounds at seven different concentrations against wild type *Leishmania Mexicana* (*Lm*) and identified ZINC20431893 (E), a saccharine derivative that interacts with the Lys335 residue of *Lm*PYK enzyme [177].

### 4.3. Molecular Target-Based HTS (MT-HTS) of the Synthetic Molecule Library (SML)

Numerous in vitro molecular target-based HTS assays using the SML are reported to date that successfully identified inhibitors of various protein targets such as penicillin-binding protein (PBP) [178], mitogen-activated protein kinase (MKK) [179], the type III secretion system (T3SS) [180], the anthrax lethal factor (LF) [181], phosphopantetheinyl transferase (PPTase) [182], peptidoglycan O-acetyltransferase [183], telomere resolvase (ResT) [184], etc. Spicer et al. screened ~292,000 compounds against M18 Aspartyl Aminopeptidase (PfM18AAP) and identified two compounds that were also active in whole cell-based assays [185]. An ultra-high-throughput MT-HTS identified sulfonyl piperazine and pyrazole compounds, targeting lipid A biosynthesis (LpxH) and lipoproteins trafficking (LolCDE transporter complex), respectively, from a screen of 1.2 million compounds against *E. coli* based on a *Citrobacter freundii* AmpC reporter [186].

Convenient molecular biology approaches to synthesize large numbers of various proteins in pure form enable cell-free MT-HTS assays for enzyme inhibitor discovery. This approach, even if it does not work in whole cell-based assays, is valuable for identifying active chemical scaffolds for medicinal chemists to work with [187] or to understand important cellular and biochemical pathways with which the inhibitors are interacting [188]. To address some of the associated challenges of HTS assays such as the identification of previously identified hits, generally cytotoxic agents, lack of activity in the whole cell assay for hits identified in MT-HTS or vice versa, failure in animal models, difficulties in determining enzyme target, etc., traditional HTS approaches have been modified and multiplexed to utilize all potential holds in drug discovery, such as testing multiple targets or multiple compounds in a single well followed by a secondary screening to confirm hits [189]. Table 4 enlists various HTS approaches utilizing the SML against different pathogenic bacteria and/or enzymes.

### 4.4. Other Miscellaneous HTS Assays Using the Synthetic Molecule Library (SML)

Lacriola et al. reported four compounds by screening 10,080 compounds based on beta-Gal release that induced autolysis of the cell membrane of *Bacillus subtilis* and also showed activity against *S. aureus*, *Enterococcus faecium,* and *Bacillus anthracis*, with MICs in the range of 12.5 to 25 μg/mL (20 to 60 μM) [197]. Langsdorf et al. reported an HTS mass spectrometry assay to determine the target of UDP-3-O-(R-3-hydroxymyristoyl)-N-acetylglucosamine deacetylase (LpxC) by measuring the catalytic activity of the enzyme by screening 700,000 compounds, which were later confirmed by a thermal stability assay and whole cell antibacterial activity against *E. coli* [198]. Rajamuthiah et al. reported an HTS imaging screening using *Caenorhabditis elegans* and found 27 clinically used antibiotics that increased the survival of the worm against *S. aureus,* which they also confirmed in vancomycin-resistant *S. aureus* isolates and other gram-positive bacteria [199].

### 4.5. High-Throughput Synthetic Molecule Library Screening against Quorum-Sensing and Biofilm-Forming Bacteria

Quorum-sensing pathways in gram-positive bacteria, such as *Streptococcus* sp., contain specific transporter systems, e.g., ComA-like transporters that contain a unique peptidase domain only found in prokaryotes, and such targeted screening minimizes finding agents that will be generally toxic to other cells, such as human cells. Additionally, as bacteria use this pathway as a communication tool, scopes of resistance development might also be lower compared to antimicrobials targeting other pathways [200]. Gatta et al. screened a 2000 compound library against *LsrK*, which is a kinase that participates in processing a quorum-sensing mediator named autoinducer-2 in gut enteric bacteria, and identified two hits: harpagoside (F) and rosolic acid (G) [201]. Screening existing chemical libraries for quorum-sensing inhibitors, a virulence-regulating signaling mechanism that promotes host defense by making pathogenic bacteria avirulent or making the host environment adverse for bacterial survival is another smart strategy to not introduce resistance during screening and drug discovery initiatives [202]. Sully et al. identified savarin (H), which inhibits *S. aureus* signaling by the quorum-sensing operon, *agr*, without affecting the other microbiota in murine models that do not have this operon [203]. Ishii et al. reported an HTS of 164,514 compounds against *S. mutans* and reported six compounds that inhibit the *MuPEP1* activity, biofilm formation, and growth of not only *S. mutans* but two other Streptococcal bacteria, *S. pneumoniae* and *S. oralis* [204].

Biofilm-forming pathogenic bacteria is another area that can be benefitted from HTS-based antibacterial screening to help prevent acute clinical infections as explored by Torres et al., who screened 1280 compounds against planktonic *S. aureus* and identified niclosamide (I), carmofur (J), and auranofin (K), which they also confirmed against preformed biofilms [205]. Shella et al. reported the identification of an anti-biofilm compound, ferutinin (L), and a quorum-sensing inhibitor with bactericidal activity, fingolimod (M), an FDA-approved drug indicated for multiple sclerosis from an HTS of libraries of natural products, and FDA approved drugs using *S. aureus* and *P. aeruginosa* as target organisms [206,207].

### 4.6. High-Throughput Synthetic Molecule Library Screening Using Biomimetic Conditions

**Figure 10:**
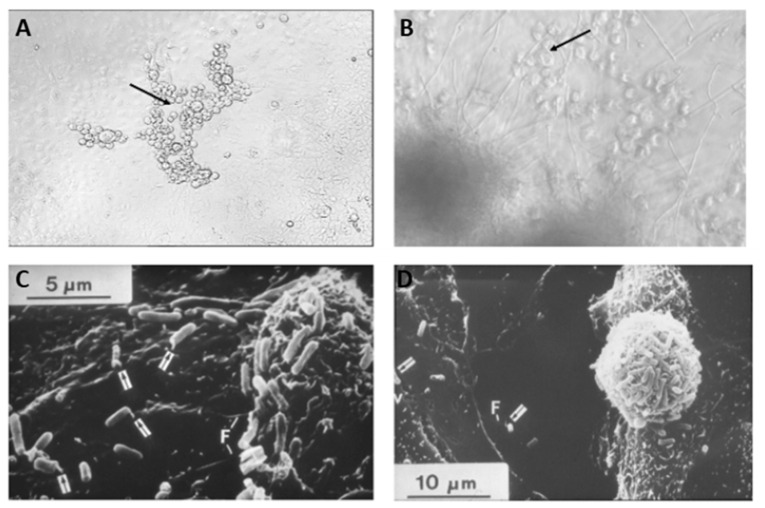
The smallest unit of a natural infection. (**A**) Microscopic picture (100×) of a viral plaque (Herpes simplex virus [HSV]) in a Vero cell culture. HSV-infected cells are lysed within 12–18 h post-infection leaving holes (arrow) in a mono layer of Vero cells after ~48 h (diameter of a single Vero cell~10–15 μM). (**B**) A light microscopic picture of a HeLa cell culture (diameter of a HeLa cell~10 μM) overgrown by *Candida albicans* (ATCC 10231) 72 h post-infection (Candidiasis [yeast infection] magnification 100×). The remaining detached HeLa cells are indicated by an arrow. (**C**) A scanning electron microscopic picture of cell culture (Vero cells) infected with bacteria (*E. coli* WF96). Bacteria outgrow eukaryotic cells. An increasing number of bacteria attach to the surface of Vero cells. (**D**) Heavily colonized epithelial cells detach from the culture dish and the mono layer is destroyed. Arrows indicate *E. coli* attached to the microvilli of Vero cells [215]. (Reprinted with permission).

Due to a lower frequency of hit-to-lead optimization, scientists have found intelligent strategies to enhance the chemical diversity, mimic the in vivo conditions, and exploit the natural microbiome for HTS. Especially, the host environment where the bacteria stay to keep their metabolic, biochemical, and virulence processes active is different than standard microbiological media, and scientists have been looking into other growth conditions that are closely related to where pathogenic bacteria live and multiply. This has the potential to help explore uncharted targets essential for their growth, survival, and infection within the host. Weber et al. used human blood serum as an alternative growth medium to explore new antibacterial agents against *Klebsiella pneumoniae* using genetic and chemical libraries and showed that serum helped to reveal cryptic antibacterial activity that required in vivo metabolic conversion, such as Prontosil [208]. Studies by Colquhoun et al. compared the HTS of an FDA-approved library against *Acinetobacter baumannii* in both standard laboratory media and human serum and reported that nine out of ninety compounds were only active when human serum was used as growth media [209]. Similarly, other biomimetic conditions, such as artificial urine to screen for uropathogens [210] and iron-depleted media for bloodstream or wound infections [211], have also been reported. Umland et al. reported the use of human ascites in identifying 18 genes in *Acinetobacter baumannii* that are involved in metabolic, signaling, DNA/RNA synthesis and regulation, protein transport, and structural functions, all of which were absent when the bacteria was being grown in rich laboratory media [212]. Enzymes encoded by these genes have never been tested as targets for any FDA-approved drugs or drugs that are in the developmental pipeline and can be potential targets for antibacterial drug discovery. Zlitni et al. demonstrated the utilization of minimal media that was devoid of vitamins, nucleobases, and amino acids to identify the mechanism of action of identified anti-*E. coli* compounds being inhibitors of glycine, PABA, and biotin biosynthesis obtained from a phenotyping whole cell screen [213]. Ellis et al. used a macrophage-mimicking media and performed HTS against *Salmonella enterica* serovar Typhimurium (S. Tm) within macrophages and identified metergoline (N) as a hit that acts by disrupting the cytoplasmic membrane [214]. They also reported that potentiation of gram-positive targeting antibiotics probably occurs due to a macrophage-induced loss of the outer membrane. Kleymann and Werling designed a screening to mimic the natural infection by incubating host cells with test compounds and pathogenic microbes, such as viruses and bacteria, and analysed the host cell survival instead of the growth of the microbes or the inhibition of any enzymes (Figure 10) [215].

This study not only addresses some of the challenges of the cellular or molecular target-specific HTS but also provides an early filter to distinguish antimicrobial agents that are not just generally cytotoxic and can help in their early elimination, which will not be viable drug candidates considering selectivity to pathogens and tolerability to host cells. Fahnoe et al. developed a similar HTS assay and tested 149,624 molecules against *P. aeruginosa* and discovered eight inhibitors of glyoxylate shunt, which are required for their survival in the pulmonary environment and were missed during traditional HTS assays [216]. Nakatsuji et al. isolated coagulase-negative *S. aureus, Staphylococcus epidermidis, and Staphylococcus hominis* from healthy patients and patients with atopic dermatitis and identified antimicrobial peptides that upon reintroduction to human subjects with atopic dermatitis showed reduced colonization [217]. With modern-day automation, such a system can be automated and holds tremendous potential in antibacterial drug discovery. However, a universal growth condition that reflects the in vivo infection within the host environment is yet to be discovered, and finding this can be very challenging as the host environment is regulated by different factors, including innate and adaptive immunity, in addition to the individual differences in the genetic composition, nutrient availability, and disease conditions.

### 4.7. High-Throughput Synthetic Molecule Library Screening, Drug Repurposing, and Synergy

Strategies for performing HTS using the SML are being extended to repurpose drugs, resensitize pathogens, and identify synergists. Cheng and coworkers screened 4096 drugs and bioactive compounds against a highly virulent, multidrug-resistant *Acinetobacter baumannii* strain AB5075, which was isolated from a patient that was found to be resistant against 25 first-line antibiotics for gram-positive bacteria. They identified forty-three hits, out of which seven were approved drugs for non-antimicrobial indications, and three molecules, namely 5-fluorouracil (O), fluspirilene (P), and Bay 11-7082 (Q), which could resensitize this notorious strain to the synergistic action of azithromycin and colistin [218]. Miller and coworkers also demonstrated the utility of drug repurposing by screening 1.6 million small molecules, which is based on a protein kinase inhibition, and they identified a series of antibacterial pyridopyrimidines that target the bacterial ATP binding site and the biotin carboxylase subunit of acetyl–coenzyme A (CoA) carboxylase (ACC), an enzyme catalyzing the first step of fatty acid biosynthesis, which showed in vitro and in vivo activity against *Haemophilus influenza* [219].

The microsomal metabolism of chemical libraries to probe drug metabolites for antibacterial activity in the presence and absence of other antibiotics was attempted by academic labs, including our group [220,221,222]. This comparative approach of screening parent and metabolite(s) of drugs and xenobiotics led to the identification of anti-Klebsiella pneumoniae agent, 6-Aminoindole Warhead from MAC-0321397 [208], and anti-MRSA agents, 5′-Deoxy-5-fluorocytidine (R), doxifluridine (S) from capecitabine, methyl balofloxacin (T) from balofloxacin, and hydroxy gatifloxacin (U) from gatifloxacin [220,222,223]. This approach identified compounds that inhibit the replication of plasmid responsible for resistance [224] or compounds that act synergistically with an antibiotic against which the bacteria are resistant [220]. Such an approach can help to revive the utility of old antibiotics by conferring the resensitization of pathogens and to explore adjuvants or synergistic molecules [225]. Several synergistic combinations have been reported from HTS assays against various pathogenic bacteria and are listed in Table 5. Synergy testing is very amenable and realistic for HTS setup as it can help test various compounds in various combinations simultaneously and conveniently. Combining hits with known antibiotics can also help determine the mechanism of action of the unknowns as reported by Farha and Brown, who identified the targets of 186 unknown bioactive molecules from a 50,000-compound screening using a synergy test with 14 known antibiotics in 2604 pairwise combinations as folate biosynthesis and a DNA gyrase inhibitor [226].

### 4.8. The Library of Synthetic Peptides and Polymers and Antibacterial High-Throughput Screening

The HTS of antimicrobial peptides and polymers (AMPPs) alone or in combination with chemical libraries has been pursued due to the large chemical repertoire they offer to target various cellular processes. They offer low cost of synthesis and automation adaptability and can be considered for drug repurposing and exploring synergy [242,243,244,245,246]. Chen et al. used a combination of high-throughput library screening with atomistic computer simulations to design a library of 2916 peptides based on the LDKA template and discovered nine peptides with minor differences in sequences but unique functional properties with an ability to form different pore sizes and varying selectivity against neutral or anionic lipid bilayers [247]. Xie et al. developed a ribosome display system that forms the peptide–ribosome–mRNA complex in vitro from nucleotides along with immobilized model membranes to identify sequences that recognize and selectively bind to only bacterial membranes, which they claim work better than a phage display system and with reduced mammalian cell toxicity [248]. Jiang et al. synthesized a 215-member mono- and diamino acid peptidic aminosugar library, which they tested against 13 bacterial strains and 27 base models of 16S ribosomal A-site RNA, and they identified a compound that can selectively differentiate a bacterial 16S ribosomal A-site from the human A-site [249]. Judzewitsch et al. developed a high-throughput flow-mediated synthesis of polymers and reported terpolymers of acrylate and acrylamide monomers that showed antimicrobial activity against *P. aeruginosa* [245]. Hilpert et al. reported an HTS to determine analogs of bactenecin that showed broad-spectrum activity against *E. coli* and *S. aureus* with an MIC range of 0.5–2 µg/mL [250]. Repurposing existing chemical libraries for antibacterial screening by probing them in the presence of metal ions, such as copper, has also been attempted and found to be effective in finding metal ion-dependent inhibitors [251,252].

Leveraging HTS technology and automation, antimicrobial peptides of diverse random sequences, sizes, and structures can now be screened for antibacterial activities using a technology known as Surface Localized Antimicrobial Display (SLAY), and Tucker et al. identified 13,600 synthetic peptides from an 800,000 20 mer peptide library as hits [253]. Cyclic peptides, on the other hand, have better pharmacokinetic properties and often elicit better bioactivity because of their conformational rigidity [254]. Depending on whether the molecules are of a biological origin or synthetic, there are various techniques for generating cyclic peptide libraries for HTS such as phage display [255], split-intein cyclization of peptides and proteins (SICLOPPS) [256], and mRNA display [257], among others. Such approaches can help to enrich our antibiotic arsenal and provide new chemical scaffolds to fight multidrug-resistant bacterial infections.

A very interesting perspective to look at the HTS of synthetic molecule libraries is that all these different approaches to HTS for antibacterial drug screening are also employed for the cytotoxicity assay in parallel, and can help to prioritize molecules or their sources before going to great lengths to characterize them [258]. Such initiatives can relieve the burden of large-scale in vivo studies and can be taken into consideration before undertaking animal studies.

## 5. Technical Considerations for Designing High-Throughput Screening Assays for Antibacterial Drug Discovery

Depending on the type, target, and goal of HTS assays, their design, specifically the assay conditions, is geared toward achieving maximum automation and throughput. For example, for a cellular target (whole cell)-based HTS assay, the assay condition depends on the type of pathogen, its physiology, growth conditions, optical output, reagents, etc. For a molecular target (protein target)-based HTS assay, the assay condition depends on the nature of the target and the condition it needs to be in to study the interaction of library molecules with the target. However, for both types of HTS assays, high-performance liquid handling robotics, an appropriately chosen library, a robust analytical system, and an efficient data management system are very critical. Although there are some assay-specific variations, most traditional HTS assays require some degree of miniaturization to enable screening of a library of compounds in multiwell plates, the activity of which is generally evaluated by analyzing some sort of optical readouts or other qualitative or quantitative measurement. A comprehensive discussion of these topics can be found in the book by Taosheng Chen [259], and some of the critical aspects are discussed below.

### 5.1. Library Selection

The chemical space comprising drugs and drug-like molecules is extremely large, with an estimate of 26.4 million to 28.8 billion, depending on the number of atoms up to 15 [260]. There are various designs for chemical library preparation such as ones that are based on diversity, known pharmacophore (focused library design), the binding mode of various chemical scaffolds (hinge binding, DFG out binding, invariant lysine binding for kinases), etc. The probability of a molecule that has structural similarity to a bioactive molecule (chemical descriptors) compared to a molecule that has activity against a druggable proteome (biological descriptor) is lower, and the latter can be a better way to expand diversity for finding hits with a greater potential to lead optimization [261]. The careful selection of molecules from this vast chemical scaffold islands is navigated in guidance with Lipinski’s rule of five, which is based on oral bioavailability; however, later studies suggested that the discovery of novel chemical scaffolds with the potential for lead optimization needs flexibility and sufficient space [262]. Features such as solubility, molecular weight, stereocenters, the number of hydrogen bond donors and acceptors, the number of rings, the number of freely rotatable bonds, logP value, average polar surface, protein binding, aggregation features, hemolysis, etc., can greatly affect the usability of the tested compounds, and various groups performing HTS generally agree on the importance of these parameters for the likelihood of successful identification of hits. Protein binding characteristics can greatly diminish in vivo activity of antibacterial agents. Compounds that tend to aggregate often do not test positive during in vitro testing. On the other hand, compounds that precipitate out or lose solubility in order to enhance bioactivity or pharmacokinetic features are rendered useless. Bael and Holloway made a recommendation about the functional groups that are unsuitable for library screening campaigns including halides, sulfonates, anhydrides, peroxides, isocyanates, triflates, positively charged carbon/halogen/phosphorus/sulfur, any heterohalide, carbodiimide, acyl cyanides, sulfonyl cyanides, disulfides, thiols, epoxides, aziridines, betalactones, betalactams, labile esters, aldehydes, certain imines, phosphate/sulfate/phosphonate/sulfonate esters, reactive Michael acceptors, ketenes, oxoniums, carbamic acids, boronic acids, primary hydrazines/oxyamines, cyclohexadienes, activated sulfonyl (hetero)aryl halides, fluoropyridines, and nitros, and these exclusion criteria were based on various parameters, such as the Tanimoto coefficient, a simple but reasonable way of excluding numerous similar analogs with popular chemistries, which is now publicly available to use as a structured filter for HTS assays [263]. However, it has also been demonstrated that antibacterial drug screening should be allowed to have the flexibility for some of these rules, as proved by the discovery of daptomycin, cyclosporine A, etc., that have higher molecular weight, and this should be kept in mind during the selection of the library, especially against gram-negative bacteria [264]. Hence, the first and foremost factor to consider for HTS assay design is the selection of the right library fit for the goal of the assay.

Initial screening concentration is another very important factor because a negligently selected testing concentration can generate too many or too few hits. This can be evaluated by testing a smaller library once other screening conditions are optimized prior to the actual screening [265]. It has been recommended to test the natural product library at two different concentrations 10–100 folds apart whenever it is practical to do so to minimize matrix and interference effects, especially for cellular target-based HTS [82]. The number of compounds screened is another important parameter for HTS assays. Statistically, the higher the number of natural product extracts or compounds tested, the higher the chances are to find a hit. For example, The Scripps Research Institute’s microbial strain collection contains a total of 217,352 bacterial and fungal strains, of which 62,328 are actinobacteria, 14,465 are other bacteria, 92,225 are fungi, and 48,334 are unidentified (bacteria or fungi) that were isolated in the last eight decades from 109 different countries where the climate, ecology, evolution, and environmental cues allow capturing a wide chemical and biological diversity [107]. Estimating ~30 biosynthetic gene clusters per strain, this collection could encode more than 6 million BGCs, with the potential of producing more than 6 million natural products. For the HTS of NPL, diversity that can greatly compensate for the number is critical. A smaller but very biodiverse natural product library has a higher chance to provide novel chemistry or bioactivity than a larger but sparse one. Cheminformatics can also greatly help in selecting a library for a specific target or disease in mind [265]. As not all strains of natural sources will make secondary metabolites for successful drug discovery, the selection of natural products and collections requires careful phylogenetic study and preparation to allow for the inclusion of diverse yet promising strains.

Library diversity enhancements based on fragment-based screening, DNA-encoded libraries, diversity-oriented synthesis, etc., are explored, and it can be realized that the selection of the library size and diversity is also dependent on technical and financial feasibility and scientific reasoning, and hence can be greatly benefitted by a prior in silico screening [262]. Diversity in chemical space can also be brought in by including various growth conditions and genetic manipulation [266]. Although a higher number of molecules or extracts is generally desired, screening a big library, such as the one from Scripps, may yield redundancies and the issue of dereplication. Mass spectrometry-based metabolomics along with ventures such as the Natural Product Atlas [267], the Dictionary of Natural Products, AntiBase, MarinLit, GNPS [268], or any in-house database search are being used in the community, which can greatly derisk the dereplication phenomena and can further be benefitted by the integration of function-based profiling platforms such as Compound Activity Mapping [158] or BioMAP [159]. Steele et al. also reported two approaches while selecting a library for HTS assays, namely (i) the structure-centric approach that prioritizes privileged strains based on a unique pharmacophore or scaffolds using genomics information and (ii) the function-centric approach that prioritizes strains based on unique targets or mechanisms of actions using biological activity information. The selection of a library should thus be diligently considered based on the aim of the study but also should be realistic from the infrastructure point of view.

### 5.2. Logistics and Technology Platforms

The success of an HTS initiative for drug discovery mainly depends on how fast and reliably a large number of molecules can be screened with the least number of materials against a panel of targets, which is greatly dependent on high-performance robotics and automation. The general HTS workflow consists of making copies of libraries in microwell plates (96, 384, 1536, or 3456 wells) from deep-well source plates, adding different reagents followed by measuring changes in each well by some sort of optical measurement (absorbance, fluorescence, luminescence, colorimetry, etc.) using multimode plate readers [269,270,271] or cell imaging [272]. Alternative responses such as measurements of cell health, metabolic state, protein expression, and the kinetics of product formation have been reported to be used for HTS assay result evaluation and hit selection [273]. During HTS campaigns, assay development, data analysis, and hit validation take more time than the actual HTS assay, and expenses are often increased due to consumables and reagents and not due to the library or automation technologies. Interestingly, it has been brought to attention that ultimate miniaturization, i.e., the use of 1536 microwell plates or higher does not necessarily reduce reagent costs, as they might require similar or more reagents to acquire better data [274]. The performance of the liquid handlers also depends on the viscosity and volatility of the solvents that can cause variation and modern instruments, such as the Labcyte Echo acoustic handler or the pressure-sensing liquid handler from Hamilton, which can avoid these errors and provide higher precision and accuracy leading to a robust assay. (Please note that the author is not recommending any particular vendor and the given examples are used for the purpose of the contents.) Whichever way is taken, the throughput and success greatly depend on the speed and precision of HTS technology [275].

Optical readout can be a complicating factor in confidently evaluating the bioactivity of colored compounds, compounds that interfere with or reduce optical measurement, or compounds that aggregate. In order to address these issues, several optical measurement systems, mock readouts of the compounds for baseline readouts, dose-dependent assays, satisfactory signal-to-noise ratios (generally at least 5:1), a good z-factor (>0.5), a low coefficient of variation (<20%), etc., should be assessed. Readout technologies are available and can read the whole plate at once, taking as little as 30 s. To avoid interference from compounds present in natural product extracts, screening under red-shifted assay conditions and chemiluminescence over fluorescence have been recommended [69]. The presence of colored compounds, instrumental errors in transferring and reagent addition, false positives resulting from compound aggregation, the presence of promiscuous highly reactive compounds, and signal interference can reduce the performance of the HTS. Some of these issues can be handled in a number of ways such as adding non-ionic detergent [276], using an HRP-PR assay as a counter-screen to identify agents that oxidize cysteine or other critical amino acid side chains of protein targets [277], changing the reactivity of compounds to be tested, such as a reduction in amide to less reactive amines [278], Hill slope analysis to identify chemical aggregators followed by the addition of inert excipient such as glutathione, albumin, casein, gelatin, etc., to neutralize their effect [279], prescreening of the library at a concentration at which it will be screened for activity in a commonly used buffer [69], going against the flurophore/chromophore [280], etc. Fiala et al. developed a photoantimicrobial HTS assay based on 24 LEDs and validated it with common sensitizers, i.e., curcumin, phenalenone, rose bengal, a hypericum extract, and methylene blue, and showed that light is a cofactor, ignoring what can lead to false negatives in antimicrobial HTS assays [281]. For fluorescence measurement, the excitation and emission wavelength should be optimized along with incubation time, which varies depending on the dye and the bacteria [282]. Luminescence was found to produce a strong signal within 24 h with a >1000-fold signal-to-noise ratio, thus reaching detector saturation faster compared to fluorescence requiring up to 72 h for yielding maximum intensity with a 10–100-fold signal-to-noise ratio in an HTS assays against *M. abscessus,* indicating that the optical output measurement should be carefully optimized for high-quality experimental data [265]. Primary screening can be conducted at various concentrations across four log ranges to construct a concentration–response curve (CRC). Screening can be conducted for a regular interval, e.g., recording data after 24, 48, and 72 h to capture both potent fast-acting hits, as well as less potent slow-acting hits.

Microplate selection (number of wells, shape, and color) should be carefully considered, as the material of the microplate has to be compatible with the solvent used. For example, polystyrene is susceptible to certain organic solvents and polypropylene can cause high UV background. The color and shape of the wells are also important; a black well flat bottom microplate can help to minimize optical interference. The direction of reading the optical output is another important feature. The solid bottom plates are better if reading from the top, whereas, if reading from the bottom, then a black wall with a clear/transparent bottom is better.

With tremendous advancements in automation technology, miniaturized HTS workflow with high precision, reliability, and human-independent operations are now available, which include a random access online compound library carousel, plate handling and transporters, lidding and delidding systems, a multifunctional reagent dispenser, and a pin array for rapid transfer and scheduling software, as well as storage, incubation, centrifugation, and wash stations [283,284,285] along with plate shakers, centrifuge, plate readers, and any other equipment for virtually any unit operation [80]. Validating the performance of HTS assays is essential, and Michal et al. discuss how they used the Library of Pharmacologically Active Compounds (LOPAC) to perform system validation using statistical parameters such as the signal-to-background ratio, Z-score, and the coefficient of variation [275]. A similar approach can greatly benefit evaluating system performance before conducting the real screening experiment, which can save time down the road and increase assay robustness. Moreover, the cost of in vivo studies with the potential risk of a lack of activity in animal models and the unpredictable correlation between animal toxicity and human toxicity profiles [286] have inspired scientists to invent 3D models as a secondary screening followed by HTS assays and an alternative to in vivo screening. However, the use of 3D models has mostly been seen with tumors [287], bones and cartilage [288], and spheroids [289], and their use in antibacterial HTS has not been reported yet and could be a great way to bridge the gap between traditional HTS and animal studies.

### 5.3. Storage and Stability

The storage of libraries is a critical step for HTS assays to ensure stability and to avoid obtaining false negatives due to compound degradation. It is recommended to confirm the hits from a different source, e.g., from a commercial source whenever available, as the source library compounds are prone to degradation due to freeze/thaw, hydration, etc. A lack of orthogonal confirmation can greatly affect the reproducibility of HTS assays and lead to the generation of false negatives. Handling and managing big libraries (>10,000 compounds/extracts) with digitized systems, such as barcoding, can be a challenging task for smaller academic labs. The temperature (−20 °C vs. −80 °C), solvent (DMSO, DMF, water, glycerol), the number of freeze-thaw cycles (as few as possible) of the source library, etc., are crucial parameters to be mindful of to achieve a robust and reproducible HTS assay. The freeze-thaw cycle can cause up to 48% degradation over a period of 1 year [290]. Routine spectroscopic and spectrometric analysis can help to minimize issues arising from stability; however, it can be challenging, depending on the resource availability. Synthesizing hits for follow-up studies can be a hurdle if a medicinal and organic chemistry skillset is absent in the HTS team and may create a challenge when the commercial source of hits for validation is not available. Other things to keep in mind during handling the libraries include not opening/unsealing library plates when frozen until they are equilibrated at an ambient temperature to maximize the shelf-life of the compounds, and short spinning before liquid transfer to minimize error in transferring volume.

### 5.4. Microorganisms and Culture Conditions

Target organisms and strain selection are crucial before undertaking HTS assays for antibacterial drug discovery. Pathogens from BSL2 and the above category require dedicated lab space, and a biosafety hood with proper ventilation and care should be taken while handling such organisms. Multidrug-resistant and biofilm-forming pathogenic bacteria resilient to both antibiotics and the host’s immune system should be handled with caution using higher safety measures [291]. Studies conducted by O’Toole and coworkers reported the use of non-pathogenic bacteria (*Mycobacterium smegmatis*) as a surrogate in vitro model for identifying hits against pathogenic bacteria (*Mycobacterium tuberculosis*) with 50% success [292]. They also reported using a drug-tolerant non-growing culture of *M. tuberculosis* for initial identification, which they later confirmed against multi and extensively drug-resistant clinical isolates [293]. The non-growing population of pathogenic bacteria, also known as persisters, such as staphylococcal biofilms, *P. aeruginosa* isolates from lung infections, and tuberculous granulomas containing latent *M. tuberculosis,* etc., have not been studied extensively as targets for traditional antibacterial screening campaigns, but attaining tractions in developing novel approaches as can be seen in the discovery of pyrazinamide that exclusively works against non-replicating bacteria by depleting membrane energy [294]. Cho et al. reported an HTS assay for identifying compounds against non-replicating *M. tuberculosis* using a luminescence-based low-oxygen recovery (LORA) assay [61]. The difference in the genome between native and pathogenic strains of the same bacteria is an important factor, as the pathogenic strain might have a specific sequence that is important for providing the right spatial arrangement for binding to the ligands, and this phenomenon has been observed with the identification of inhibitors of various molecular targets such as RNA polymerase in *S. aureus* [295], acetohydroxyacid synthase in *M. tuberculosis* [296], shikimate dehydrogenase in *H. pylori* [297], etc.

The culture condition is another critical factor to consider during the designing of antibacterial HTS assays, and the O’Toole group reported that some inhibitors, such as Se-methylselenocysteine, were only detected when grown under nutrient-limited culture conditions as opposed to nutrient-rich culture conditions [298]. By including host-related factors or mimicking the actual host environment, alternative antibacterial compounds could be detected, as also showed by Weber et al. [208], and can help to correlate results from in vitro assays with in vivo studies [299]. Gordhan et al. discussed different model systems to study the adaptability of *M. tuberculosis* when exposed to various stress conditions such as hypoxia, carbon starvation, nitric oxide exposure, acid exposure, antibiotic exposure, etc., which put bacteria into a non-replicating state from which they can come back when they are adapted to the stress or when the stress has been removed [300]. Additionally, care should be taken to select the right media condition based on the type of measurement used in the HTS assays, as ingredients present in media such as salts, metal cations, etc., can interfere with the optical readout.

The final percent of organics in each well of the multiwell plate is another critical factor to consider when allowing sufficient solubility of the screening molecules and at the same time maintaining a permissible environment for bacterial growth. Often, DMSO is a preferred universal solvent for library compounds to maximize solubility and stability (faster freezing); however, it can hinder bacterial growth if present at a higher percentage. Our experience with MRSA ATCC 43300 demonstrated regular growth in the presence of up to 2% of DMSO, which also corroborates with the DMSO tolerability of *Staphylococcus aureus* ATCC 29213, *Salmonella typhimurium* ATCC 14028, *Klebsiella pneumoniae* ATCC 4352, *Acinetobacter baumanii* BAA-1605, *Pseudomonas aeruginosa* ATCC 27853, and *Escherichia coli* ATCC 25922, all of which showed tolerance of up to 2.5% of DMSO [234]. Gupta et al. also reported tolerability of *Mycobacterium abscessus* of up to 2% of DMSO and 10% of acetone, which, however, encountered up to 20% growth inhibition in the presence of 0.5% DMF [265].

The number of cells per well and the growth phase they are in are also important and should be optimized. Growing a primary bacterial culture (exponential or stationary phase), diluting with fresh media to make a secondary culture, and then using the latter for inoculation can reduce the risk of failed assay due to growth problems [206,282]. The growth of bacteria in microwell plates should be assessed for different incubation times by studying the growth kinetics, as it will vary with bacterial strain, culture volume, type of plate, and buffer used [171]. Gupta et al. tested different optical densities (0.01, 0.05, and 0.1), different volumes (30, 50, and 70 µL), and different incubation times (24, 48, and 72 h) of the secondary bacterial culture, i.e., starting inoculum, and reported excellent results with cultures with an OD_600_ of 0.01 irrespective of culture volume and incubation time with a Z-factor of 0.8 for *M. abscessus,* which was probably due to lower cell clumping [265]. Needless to say, a panel of carefully selected positive and negative controls (antibiotics) should always be tested both during the optimization and experimental phases. It should also be kept in mind that optimization performed in an agar-based assay might not translate into liquid screening; hence, optimization should reflect the real HTS conditions [199]. Other factors that can affect bacterial growth such as aeration, pH, temperature, humidity, shaking and shaking speed, etc., should be carefully monitored and optimized. Unfavorable growth conditions in microwell plates will affect the growth of the bacteria whether they are the target organisms or whether their extracts are to be tested as the source of new bioactive molecules.

**Figure 11:**
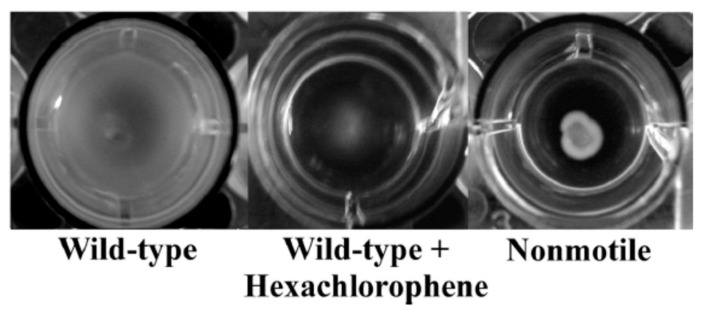
Images showing three types of growth patterns observed for the center of *Salmonella typhimurium* cells inoculated in the larger wells of a 24-well tissue culture plate. (**Left**) Wild type SJW1103 cells in a plain Luria–Bertani (LB) agar. (**Center**) Wild type SJW1103 cells grown in the presence of 30 μM hexachlorophene showing the partial inhibition of growth and motility. (**Right**) Non-motile SJW134 cells grown in a plain LB agar [172]. (Reprinted with permission).

Bacterial motility is important, especially for the biofilm-forming bacteria, and light swirling should be used to help bacteria be evenly distributed in the liquid culture [38], which will also be directly reflected in the optical readout. Biofilm-forming pathogenic bacteria leads to acute infections in clinics, as they have the ability to form biofilms as a shield to stress and/or antibiotic exposure. Malapaka et al. discussed the motility characteristics of different strains of *Salmonella typhimurium,* and it is important to assess and differentiate a hit from a non-hit [172]. This should be specifically considered when the target bacteria are non-motile (Figure 11).

Hence, it is good practice to briefly spin the microtiter plates after primary inoculation and before measuring optical readout, especially if the absorbance of the turbidity is measured. Gentle shaking during the incubation of the library with the target organism should be assessed during assay development and optimization. The growth pattern is another important factor in antibacterial HTS design, especially for bacterial subpopulations that are auxotrophic, such as *Staphylococcus aureus* small-colony variants (SCV) [301], and can lead to more serious chronic infections [302], as antibiotics of various class including aminoglycosides, cationic compounds, antifolates, cell walls, and protein biosynthesis inhibitors have less activity against SCV [303]. For these types of slowly growing bacterial subpopulations, an HTS assay based on adenylate kinase (AK) release can be used, which is a ubiquitous intracellular enzyme that is released when the cells are lysed and can be measured to assess the potency of the tested compounds [304]. An AK-based HTS approach has been reported to screen antibacterial agents against *Staphylococcus aureus* [304] and *Pseudomonas aeruginosa* [305]. However, AK-based HTS depends on the lysis of the cells and can vary depending on the specific SCV strain tested, as they are heterogenous subpopulations with varying auxotrophism, which will directly impact the results. In order to screen for viable but not culturable strains, there are various techniques to select from, including fluorescent and nucleic acid dye, for checking membrane integrity, membrane potential and ATP measurement, respiration measurement, viability PCR, nucleic acid sequence-based amplification (NASBA), the molecular viability test (MVT), stable isotope probing, etc.; the details of which can be found elsewhere [306].

### 5.5. Orthogonal Assays for Hit Validation, Toxicity Screening, Dereplication, and Target Identification

Orthogonal assays are essential components in HTS campaigns to confirm identified hits, as well as to filter out non-specific and toxic molecules. Whether using a natural product or a synthetic molecule library, HTS assays for bioactivity often suffer from a lack of specificity, i.e., often times the hits generated turn out to be generally cytotoxic. Evidently, this is a problem, as pointed out by Dahlin and Walter in their review that PAINS or related molecules are still being reported as bioactives/hits in research articles due to a lack of information on these structures [307]. Knowledge of the history of these molecules or utilizing public databases such as SciFinder, CAS, PubChem, etc., to search for structures or substructures of these molecules can help to screen the hit list to eliminate PAINS and toxic molecules from further consideration. Prescreening libraries against a high concentration of soluble proteins that are present in culture media or counter-screening against a target that is unrelated to the primary target (such as the β-lactamase AmpC assay) can help to remove PAINS compounds in molecular target-based HTS assays [308]. Parallel screening of the SML or at least follow-up screening of short-listed hits against mammalian or human cell lines is critical to identify cytotoxic molecules to prevent investing further effort in pursuing a hit that is indiscriminately toxic.

The risk of identifying previously discovered bioactive molecules is another challenge for natural product-based HTS. To prevent the rediscovery of already existing molecules early in the HTS effort, matching the analytical data of crude extracts to a database of natural products such as the Natural Product Atlas [267], the Dictionary of Natural Products, AntiBase, MarinLit, or similar databases can help to save time and money. Being a complex mixture with potential additive biological effects, screening at multiple concentrations has been recommended to localize and isolate pure bioactive molecules and to identify nuisance compounds [309]. The difficulty in identifying the biochemical or molecular target of a hit is another challenge for which cell-based assays followed by a target-based assays have been recommended. Other approaches such as measuring enzymatic activity upon exposure to library compounds, changes in metabolome, proteome or the genome of target organism, etc., upon exposure to identified hits can also help in target identification. These approaches can be very challenging for natural product extract based screening, which is a mixture of many compounds and often contains nonspecific and toxic molecules [310].

### 5.6. Error Management, Quality Control, and HTS Triage

HTS assays generate big datasets and detecting errors in them can be a daunting task. Random errors which are generally caused by noise can be investigated by increasing replication; however, systematic errors that are caused by instrument malfunctioning in liquid transfer, reagent loss or evaporation, and protein target or compound degradation are issues with cell culture that can be difficult to investigate, as this error propagates to each replicate leading to high confidence in reproducibility. Statistical analyses such as Student’s t-test, χ2 goodness-of-fit, discrete Fourier transform (DFT) in conjunction with the Kolmogorov–Smirnov test, matrix error amendment, and partial mean polish are some of the approaches to investigate systematic errors in HTS assays [311,312]. Various software such as the JTS Navigator, HTS Corrector, HDAT, HCS Analyzer, HTS navigator, WebFlow, etc., is also available to diagnose HTS data for error check and correction [261]. A widely used statistical parameter in HTS assays is Z-factor calculation, the value of which should be above 0.5, which indicates how well the HTS assays are performing to differentiate between the actives and the inactive molecules. A poor Z-factor may result from poor or incorrect assay optimization, variation in cell or target molecule amount, contamination issues, a lack of precision during library preparation, especially by liquid handlers, etc. The cut-off for hit selection should also be carefully culled and it should be based on an appropriately selected positive control (known antibiotics/inhibitors) and a negative control (known molecules with no antibacterial activity or no target engagement) and is generally selected as three standard deviations about the mean percent inhibition (‘3σ’) [307]. In addition, during data analysis, locating and marking compounds with incompatible features for use as biological agents that are added to the library to increase the diversity can help to derisk efforts by not focusing on such nuisance compounds for further analysis. For quality control purposes, a pooled sample of a few inactive extracts of a natural product library can be used to establish the baseline matrix effect for the library and a negative control, whereas a known active compound or a set of active compounds can be used as a positive control. For synthetic molecule library HTS, a panel of known antibiotics with different mechanisms of action and antibiotics that are inactive can greatly aid in determining the robustness of the HTS, which can also help in evaluating compound stability.

HTS triage management can be greatly benefitted by a dedicated group of medicinal chemists, biologists, and analytical chemists working together and following up on the HTS campaigns closely. This intervention can occur at any step during HTS starting from library selection, assay design, hit assessment, orthogonal assays, synthesis schemes for analog studies and scale-ups, etc. [307]. Readers are encouraged to consult the best practices discussed by Dahlin and Walters for HTS designing [307]. Unless each compound from an HTS campaign has not been followed-up in greater detail, some sort of triage already took place. However, meaningful triage takes careful observation, design, and decision-making, and even after no hits may succeed in becoming a lead, but it generates critical information such as the identification of promiscuous bioactive compounds, false positives, etc., and can be incorporated into the execution of a better performing HTS assay in the future. Rutger Folmer discussed how AstraZeneca used different biophysical approaches at different stages of HTS to make critical decisions regarding undertaking or continuing HTS campaigns based on target engagement using a smaller library comprising 7000 compounds [313]. On the other hand, Dahlin and Walters discussed how they used cheminformatics to filter, sort, and rank promising core structures or pharmacophores informed by the knowledge of medicinal chemistry, physicochemical properties of the library molecules, and bioactivity data.

### 5.7. In Vivo Studies and Pharmacodynamic and Pharmacokinetic Characteristics

Antibacterial screening should not only be focused on how potent the hits are (although it may help to find agents with clinical MICs) but how safe and effective it isto use in the animal model. Additionally, from the formulation point of view, the physicochemical properties, as well as their effect on the host (pharmacodynamics) and the host’s effect on them (pharmacokinetics), should be studied carefully. Often, improving the antibacterial activities of an identified hit is found to be easier than removing the toxicity features, which is especially important to consider when the active agents are substrates of efflux pumps, and instead of killing, pathogens can accumulate in the host and lead to toxicity [314]. The importance of in vivo studies in antibacterial drug discovery is not only important to demonstrate the clinical utility and safety of the newly discovered agents, but also for discovery purposes. It has been reported that essential pathways that are being inhibited by certain molecules during in vitro assay conditions do not always result in growth inhibition during in vivo study [315]. Due to ethical and economic constraints, instead of in vivo conditions, in vivo-like conditions, such as using culture conditions that mimic the host environment more closely, could be used, and they have demonstrated their utility [299,316,317,318], as also discussed in Section 4.6. Simpler and cheaper animal models, such as *Caenorhabditis elegans,* that share a lot of genetic similarities with humans [199], amoeba, *Drosophila melanogaster*, *Arabidopsis thaliana*, zebrafish, silkworms, wax moths, etc., can also be used as alternative animal models for in vivo studies, all of which are known victims of various gram-positive and gram-negative pathogens [25,319]. Interestingly, Moy et al. reported an HTS of 37,200 compounds and natural product extracts and identified 28 compounds that cured *Enterococcus faecalis* infected *C. elegans*, and the reported in vivo effective dose of many of the identified hits was lower than the inhibitory concentrations required to prevent bacterial growth in vitro, indicating that these identified hits would be missed in conventional phenotypic whole cell/cellular target-based assays [320]. Hilbi’s group developed a fluorescence-based assay that continuously monitors the bacterial replication rate inside the host and reports their gene deletion upon drug exposure. Using this technology, they performed a series of studies using *Acanthamoeba castellanii* amoeba that hosts *Legionella pneumophila* where the latter can reproduce inside the amoeba, escape antibiotic exposure, and cause human infection [321]. They reported several antibacterial compounds, including the β-lactone-based Ras depalmitoylation inhibitor palmostatin M [321] and the protein phosphatase calcineurin inhibitor ZINC00615682 [322].

## 6. Technologies and Other Auxiliary Approaches for Antibacterial HTS Assays

HTS screening, especially molecular target-based HTS assays undertaken by many pharmaceutical companies including GSK, Pfizer, Cubists, Wyeth, Bristol Myers Squibb, Bayer, etc., resulted in very little success against spending huge resources due to various complex reasons including targets that are active in vitro but are inactive while in the host environment, the mutation of the target in a different genetic strain of the same bacterial species making active an inactive, resistance development, etc., [33] in addition to other reasons discussed earlier. A lack of success with little economic output discouraged them and many others from actively pursuing antibiotics research. However, this does not diminish the threat that infectious diseases pose, on the contrary, it emphasizes why tomorrow could be too late to search for alternatives. Many groups are working under the sun to find ways to battle this never-diminishing issue and some of these alternative approaches are discussed below.

### 6.1. Selective Screening

A virulent phage cocktail that selectively targets pathogenic bacteria and does not harm eukaryotic cells is a selective screening assay used to eliminate foodborne pathogens; however, it can only target one pathogenic bacteria at a time in the host environment [323]. Screening for prophage-inducing agents has been reported to control the growth of *Salmonella enterica* and Shiga toxin-producing *E. coli* and was found to effectively induce multiple prophages in different bacterial genres [324]. Tomkins et al. developed an HTS assay based on prophage induction that triggers beta-galactoside production in a genetically modified *E. coli* K-12 strain. They tested several natural products and bioactive collections, such as a library from the Centre for Microbial Chemical Biology (CMCB), McMaster University (Hamilton, Ontario, Canada) of 3747 compounds that contains FDA-approved drugs, off-patent drugs, natural products, and other compounds with demonstrated biological activity. Although they could not differentiate if the activity of the identified hits was due to prophage induction, this approach certainly could be a very promising avenue to explore for antibacterial drug discovery [325]. Studies directed at bacteria-specific enzymes such as N^5^-carboxyaminoimidazole ribonucleotide (N^5^-CAIR) synthetase [326], bacterial 16S ribosomal A-site RNA [249], etc., have successfully reported agents that could avoid interacting with human biochemistry and could help to find novel and unexplored pathways and enzyme targets in bacteria, which can greatly help with antibacterial drug discovery. On the other hand, targets that are not essential for bacterial growth in standard media but are essential to adapt in vivo when encountering pressure from the host immune system or are virulence factors, such as lipopolysaccharides, could help in identifying novel antivirulence compounds, as reported by Novartis while using *Tetrahymena pyroformis* in a co-culture system with *K. pneumoniae,* where they screened circa 1.2 M compounds and found a novel LpxC inhibitor with weak antibacterial activity [327]. Thaker et al. reported the use of vancomycin as an intrinsic antibiotic resistance inducer to isolate glycopeptide antibiotic producers from soil actinomycetes, which can be utilized to selectively induce resistance and manipulate biochemical pathways to isolate diverse chemical scaffold producers from various sources and can also minimize rediscovering known molecules [20]. This group also adopted the BOX PCR approach to achieve dereplication by selectively choosing signature amplicon and reported the discovery of eleven unique glycopeptide antibiotic producer strains from one hundred vancomycin-resistant strains and one novel glycopeptide antibiotic, pekiskomycin [328].

### 6.2. Genetic Engineering, Synthetic Biology, and Omics Technology

Genetic engineering allows manipulation of the biosynthetic and translational machinery of an organism leading to differential metabolomic profiles and the expression of cryptic metabolites. Screening with genetically engineered microbes was reported by Choudhary and Rao who developed a recombinant microbial expression system and generated thousands of mutants from which they identified variants of glycosylated bacteriocin (glycocins) against *L. monocytogenes* [329]. The presence of polyketide synthases (PKSs) and nonribosomal peptide synthetases (NRPSs) in the producers generally indicates the likelihood of biosynthesis of novel compounds. High-throughput identification of natural products using analytical technology and their correlation with genomics can help in identifying these novel compounds [330]. Warp Drive Bio reported mining genomes of >340,000 actinomycetes, assembled 135,000 genomes, and logged ~3.5 million antibiotic biosynthetic clusters into a proprietary database that has high promise to reveal novel antibiotics [331]. Mutagenesis of NRPS and PKS domains led to the identification of an isoleucine-containing analog of andrimid with better antibacterial activity than andrimind itself [332]. Heterologous expression of biosynthetic enzymes and the secondary metabolites from natural sources that are difficult to grow in laboratory conditions into cultivable hosts such as *E. coli*, *Saccharomyces cerevisae,* and *Streptomyces coelicolor* allowed access to organisms that were not possible to study before, e.g., marine invertebrates including sponges, soft corals, tunicates and bryozoans. Artificial chromosome and heterologous expression have enabled the identification of new metabolites and their biosynthetic gene clusters (BGCs) from *Aspergillus nidulans* [333]. Heterologous expression also enabled high-scale production of bioactive molecules, e.g., artemisinin by genetic manipulation of biosynthetic enzymes in engineered *E. coli* [334]. These efforts demonstrate the potential of genetic engineering and synthetic biology approaches in producing complex bioactive natural products in easy-to-grow host systems.

Genome mining has enabled the identification of biosynthetic gene clusters (BGCs) of secondary metabolites and gave rise to paired omics technology [335] that can enable the correlation of genomics to metabolomics data, and its utility has been demonstrated in bacteria [336] and fungi [140]. Combined omics technologies, such as metabologenomics and proteogenomics, have been successfully used to discover new bioactive natural products, phosacetamycin [337] and an orphan of an NRPS/PKS hybrid cluster, and rakicidin D [338], and helped to connect genes to bioactive molecules such as lactocillin, thanamycin, telomycin, etc., and demonstrated its utility in identifying antibacterial compounds from natural sources.

In addition to identifying new antibacterial agents, genomics-based approaches also enabled the identification of conserved genes in pathogenic bacteria that are also unique and absent in eucaryotic genomes and helped in identifying novel targets. Traditional molecular target identification has also relied on genetic engineering techniques such as cloning, expression, and purification of the target. Additionally, genomics helped to understand resistance development by pathogenic bacteria, which is also beneficial for infectious disease management. Additionally, screening libraries or promising leads against metagenomics libraries will help in identifying existing mechanisms of resistance in target bacteria that will likely induce resistance upon use [339] and will ultimately increase the antibiotic duration of utility in the successful treatment of various infections. Whole genome sequencing and metagenomics can lead to a world of new bioactive molecules and biocatalysts from unculturable strains and can help in mapping out the metabolic pathway of all organisms, which will allow synthetic biologists to make any complex molecules using various enzymes. Considering the absence of novel molecules in the chemical library and the complexity of natural product library screening, a DNA-encoded library can enable accessing underrepresented chemical space, but the success of finding novel antibacterial chemistry is still yet to be seen [340].

### 6.3. In Silico/Virtual Screening

Computer-aided virtual screening has also gained popularity with the benefit of screening molecular models against a molecular target and can be used as an informed guide to direct laboratory testing [341]. In silico screening not only can help to find chemical scaffolds that will bind to a target molecule but it can also display the interaction of the ligands to the targets [342]. The hits derived from this type of screening can then be tested in unmodified laboratory conditions or can be used as a primary scaffold to synthesize analogs to check antibacterial activity [343]. Virtual screening can also help in identifying and eliminating PAINS and other promiscuous bioactive molecules and ultimately help in identifying true hits with the proper use of time and resources. X-ray crystallography and docking-based analysis of hits from HTS assays along with the traditional molecular target-based approach can greatly help in identifying the pharmacophore [344]. Virtual HTS can either be ligand-centric or structure-based, and a number of ligand-centric approaches are pharmacophore modeling, quantitative structure activity relationship modeling, and similarity searching. With the increased availability of public bioactivity databases such as GDB (~977 million compounds), ZINC (35 million compounds), and eMolecules (~6 million compounds) [307], virtual HTS has been successful in identifying ligands/inhibitors of several protein targets including DNA methyltransferases (olsalazine, nanomycin), JAKc (diazaindazole scaffold), kinases, various receptors (GPCRs, NPY5, adenosisne, neurokinin-1, mGlu4), etc. HTS fingerprinting and bioactivity modeling demonstrated better potential in hit identification, retrieval, and the mechanism of action analyses.

Sahner et al. identified inhibitors of bacterial RNA polymerase by virtually screening 42,000 compounds and confirmed activity against both gram-positive and gram-negative bacteria [345]. Chan et al. reported the identification of a promising *S. aureus* SrtA inhibitor based on the 2-phenyl-2,3-dihydro-1H-perimidine scaffold using an advanced computer-docking methodology called the relaxed complex scheme [346]. Fung-Yi Chan and coworkers identified a new class of filamenting temperature-sensitive mutant Z (FtsZ) inhibitors containing a pyrimidine–quinuclidine scaffold by a structure-based design and in vitro screening of 20,000 compounds, which were found to be active against *S. aureus* and *E. coli* in vitro [347]. One of the biggest ultra-large library docking studies by Lyu et al. investigated the structure-based docking of 170 million compounds from 130 well-characterized reactions representing 10.7 million chemical scaffolds and identified an AmpC β-lactamase inhibitor, which was later validated using synthesis [348]. Stokes et al. used artificial intelligence to discover a new antibiotic called halicin from a pool of more than 100 million molecules that works against a wide panel of bacteria causing tuberculosis and some other untreatable infections by disrupting the flow of protons across cell membranes [349]. Hu et al. screened the NIH Molecular Library Small Molecule Repository (MLSMR), a collection of 350,000, and a ChemNavigator database containing 14 million unique compounds against a model of an FtuGabl-NAD enzyme complex and found a 3-substituted indole inhibitor that also showed antibacterial activity in whole cell screening against *Francisella tularensis* [350]. Kinjo et al. reported a structure-based drug screening (SBDS) with which they screened 154,118 compounds against the enoyl-acyl carrier protein reductase (InhA) and identified two compounds with potent activity against *M. tuberculosis*, with a follow-up screening of 461,383 compounds where they identified analogs of one of the hits with no mammalian toxicity [351]. Brynildsen et al. reported an in silico genome-wide metabolic model system that can predict targets to increase the reactive oxygen species generation by an engineered *E. coli,* which was confirmed in vitro and demonstrated susceptibility to antibiotics [352]. Dai et al. developed a computational antibiotic screening platform, CLASP, that helps to find molecules that will flux into bacteria through porins and provides thermodynamic, kinetic, and spatial information during in silico screening [353]. With the advancement of computer simulation, high-performance computing, and artificial intelligence, in silico screening can help to save costs and actual screening of compounds and provide novel scaffolds against different target proteins, as can be realized from numerous examples discussed above. Details of this HTS approach in drug discovery can be found elsewhere.

### 6.4. Combinatorial Chemistry and the Focused Synthetic Approach

Combinatorial chemistry-based approaches, such as solid-phase peptide synthesis, aid in the creation of a library of thousands of compounds bearing a shared chemical backbone [354]. Ideal combinatorial library design will provide high structural diversity while preserving suitable physicochemical properties such as solubility, geometrical shape, size, molecular weight, etc. Otherwise, a hit from such library screening may fail to be optimized as a drug due to insufficient interaction with the molecular target for not having the right spatial arrangement [72] or fail to provide bioactivity in vivo [72]. On the other hand, if combinatorial library synthesis is only focused on chemical accessibility and a higher number of species for screening without taking biology into consideration, it may fail to provide any bioactivity [355]. Combinatorial style synthesis of a library of molecules that are structurally related, i.e., analogs, can be a championing approach for antibacterial drug discovery, as finding one antibacterial agent paves the way to explore various analogs with a similar or improved activity that may enrich our antibiotics arsenal, as well as allow circumventing antibacterial resistance [72].

There are several reports of success in this route in finding hits against ESKAPE pathogens, especially by the Shaw lab at the University of South Florida, among others. Fleeman et al. conducted comprehensive combinatorial studies to screen for antibacterial agents against ESKAPE pathogens. They included 37 different libraries each consisting of between 10,000 and 750,000 structural analogs for a total of more than 5 million compounds and identified bis-cyclic guanidine analogs and a polyamine scaffold with efflux pump inhibition [233] against all ESKAPE pathogens [356]. Itoh et al. reported high-throughput synthesis of lysocin E analogs leading to 2401 cyclic peptides using a one-bead-one-compound (OBOC) strategy from which they identified eleven analogs with comparable antibacterial activity against six gram-positive bacteria, including MRSA; they also identified the pharmacophore amino acid residues that were crucial for bioactivity [357]. Ansari et al. identified 1,3-bis(2-hydroxyphenyl)prop-2-en-1-one as an antibacterial agent against *S. aureus* and *B. subtilis* using a 120-member chalcone library [358]. McClay et al. demonstrated combinatorial biocatalysis using microbe-derived enzymes to generate indole scaffolds containing analogs through enzymatic oxidation and polymerization with a broad range of antibacterial activity, especially against *M. tuberculosis* strains that are resistant to front-line anti-TB drugs [359]. Takada et al. reported a high-throughput synthesis of more than 4000 analogs of gramicidin, a peptide antibiotic that disrupts the transmembrane ion concertation gradient by forming an ion channel in the lipid bilayer, and identified one analog, which was more potent than gramicidin against five gram-positive bacteria, *S. pyogenes*, *E. faecalis*, *S. pneumoniae*, *S. agalactiae*, *L. monocytogenes,* and MSSA [360].

Like the combinatorial synthesis scheme, combinatorial biosynthesis has been explored by engineering biosynthetic gene clusters for the bioactive molecule through site-directed mutagenesis, gene deletion (daptomycin), extension (balhimycin), exchange (surfactin), point mutation to make analogs of the bioactive scaffolds, such as antibacterial natural products encoded by polyketide synthase (PKS), and non-ribosomal peptide synthetase (NRPSs) [71]. PKS and NRPSs-based combinatorial biosynthesis approaches allow the biosynthesis of metabolites by sequential condensation of short fatty acids and alpha-amino acids, respectively. This approach can help in making a suit of analogs of secondary metabolites, a lot of which might not have any known bioactivity, and the purpose of their synthesis by the source is unknown but it is predicted to be the organisms’ need to have their own repertoire of molecules. It might be worth testing the effect of perturbing different organisms with a bioactive scaffold such as yohimbine, paclitaxel, vancomycin, or other PKS and NRPS metabolites to check if they can lead to achieving analogs with similar or improved bioactivity or other drug-like molecules [361]. Waldmann and coworkers demonstrated an amalgamation of fragment-based drug design and diversity-oriented synthesis where they screened various natural product fragments to identify ligands that bind to a target protein and then used them as scaffolds to make new molecules, some of which they tested and found active against P38 MAP kinase and several tyrosine phosphatases [362].

### 6.5. Microfluidic, Nanofluidic, and Imaging-Based Technologies

Microfluidic- and nanofluidic-based approaches have been proven to be promising alternatives to traditional HTS assays for antibacterial drug discovery that enable manipulation of liquids in micro to nanoliter volumes and increase the throughput by several orders of magnitude. However, they require sophisticated instruments, precision controls, and high sensitivity readouts in spite of suffering from phenotypic heterogeneity. Originally, these assays were not developed with multiwell plate format settings, which necessitated their reformatting. Additionally, limited material availability in each sample yields sensitivity issues, and based on the droplet formation mechanism, it may require challenging techniques to extract droplet-containing biomolecules. However, it has been reported in the successful isolation of antibiotic-resistant bacteria, such as fusidic acid-resistant *E. coli* mutants in a label-free manner [363] and other resistant mutants from fecal samples [364]. This technology can serve as an alternative to whole cell-based HTS assays by not only identifying antibacterial agents but also screening them against slow-growing strains and various resistant strains. More logistics and design details of microfluidic-based HTS can be found in the review by Rienzo et al. [80]. Mocciaro et al. developed a nanofluidic device demonstrating the capability of isolating single T cells on a chip followed by clonal expansion and the selective isolation of cells based on genotypes in nanoliter volume controlled by light [365]. Imaging-based HTS, such as confocal microscopy, not only allows rapid identification of hits but also allows studying other cellular characteristics such as cell cycle, motility, morphology, receptor internalization and protein distribution, the phenotypic impact of chemical or genetic perturbations, disease biomarkers, and molecular and genetic grouping into functional pathways [366,367,368]. Such an approach has been used to find compounds that induce NF-κB nuclear translocation from libraries comprising purified natural products and small molecules with diverse structural groups [369]. These technologies hold the potential for future antibacterial drug discovery research.

### 6.6. Phage Display and Antibody-Based Technologies

A phage display is a popular target-based technology where genetically modified DNA phages are used to encode different peptides. This technique was successfully used for the detection and treatment of cancer and can be used for target identification based on protein–ligand (peptide) binding in a high-throughput manner [370]. Hits from such an assay can then be followed by whole cell and in vivo assays for antibacterial activity confirmation and safety profiles. Similarly, antibodies can bind to specific targets and prevent infections, e.g., raxibacumab, an antibody that is also derived from a phage display library reported to prevent infections caused by *B. subtilis* by blocking bacterial toxin’s interaction with mammalian receptors [371]. These approaches hold promise and can lead to a revolution in antibacterial drug discovery research.

### 6.7. Metal Nanoparticles

Metal nanoparticle-based HTS assays have also been reported to have differential activity against gram-positive and gram-negative bacteria [372] due to their high surface area and reactivity. Nanoparticles made of metal and metal oxides such as silver, copper, gold, tellurium, bismuth, zinc oxide, titanium oxide, copper oxide, nickel oxide, aluminum oxide, iron oxide, cerium oxide, silicon dioxide, etc., have been reported to have antimicrobial activity [373,374]. Jin et al. tested silver nanoparticles against *B. subtilis* and *P. putida* at different concentrations using a synthetic freshwater matrix which controls the physicochemical behavior of the metal nanoparticles such as aggregation, dissolution, reprecipitation, etc., and influences the antibacterial activities of the metal nanoparticles [372]. Furthermore, a metal nanoparticle is reported to increase the antibacterial activity of the antibacterial natural product, catechin, against *S. aureus* and *E. coli* by damaging the cell membrane [375], which shows its potential application as an antibiotic adjuvant. Most of the metal nanoparticle-based antibacterial screenings have reported utilization of traditional disc diffusion, dead time test, modern flow cytometry, or dye-based techniques [376,377]. CometChip technology was developed to evaluate the genotoxicity of metal nanoparticles in human and hamster cell lines [378], and such a platform could help to design similar HTS assays for antibacterial drug discovery research.

### 6.8. Spectrometry, Cytometry, Spectroscopy, and Other Biophysical Approaches

Analytical tools such as liquid chromatography, mass spectrometry, NMR, Fourier-transform infrared (FTIR) spectroscopy, etc., play critical roles in the purification and structure elucidation of hits obtained from HTS assays. NMR has been a very popular technique to study the engagement of a ligand to its molecular target and has been used extensively to profile the binding of compounds, i.e., hit profiling, validation, pharmacokinetic profiling, etc. [313]. FTIR spectroscopy along with machine learning has also demonstrated the accurate identification of antibiotics and their mechanism of action even at very low concentrations [379]. Apostolos et al. reported an HTS platform using flow cytometry, SaccuFlow, to investigate molecules that interact with the cell wall peptidoglycan layer that both gram-positive and gram-negative bacteria use as a barrier to entry of molecules that are a threat to them, and they identified amsacrine and D-Alanylated teichoic acid as hits [380]. Other platforms, such as X-ray crystallography and cryo-EM, have enabled studying the detailed structure of several antibacterial target proteins such as topoisomerases, RNA polymerase, aminoacyl-tRNA synthetase, peptide deformylase, enzyme-regulating peptidoglycan biosynthesis, and other enzyme-regulating metabolism and cell division that have helped to screen for antibacterial agents [381].

Recent years witnessed tremendous advancements in mass spectrometry-based research, and the ability to hyphenate mass spectrometers with drug screening workflows in a high-throughput fashion enabled not only the identification of bioactive molecules but also their molecular target [382]. A RapidFire MS system by Agilent and Echo MS by Labcyte/AstraZeneca demonstrated analyzing one sample in 10 s and 0.33 s, respectively, which can assist in the rapid identification of hits from protein-based HTS by measuring the formation of catalytic product in a label-free manner [383,384]. Self-Assembled Monolayer Desorption Ionization–Affinity Selection Mass Spectrometry (SAMDI-ASMS) [385], an Automated Ligand Detection System (ALIS) [386], and DARTS-MS (Drug Affinity Responsive Target Stability–Mass Spectrometry) [387] demonstrated the power of mass spectrometry in target identification. Mass spectrometry-based metabolomics enabled the identification of precursor and various intermediates of the bioactive molecules during their biosynthesis using a database search and can be extremely helpful in engineering pathways to increase the yield of the bioactive natural products. Mass spectrometry-based proteomics enabled the identification of the complex network of biochemical pathways that get perturbed due to exposure to an antibacterial agent and unravel the crosstalk between the genes, proteins, and metabolites [388,389,390,391,392,393,394,395,396,397,398,399,400,401]. Cummins et al. reported the binding of an aminoglycoside antibiotics paromomycin in HPLC fraction derived from *Streptococcus rimosus* by mass spectrometry analysis of the non-covalent adduct of paromomycin with a synthetic RNA oligomer containing an *E. coli* A-site using dubbed multi-target affinity/specificity screening (MASS) [402]. A very recent mass spectrometry-based untargeted metabolomics with multistage native mass spectrometry has been developed by Nguyen et al., which demonstrated the binding of ligands to a human protein target by one-step direct screening of natural product extracts containing thousands of drug-like molecules [403]. Similarly, Lomenick et al. demonstrated the metabolite–protein interaction using mass spectrometry, a technique they named drug affinity responsive target stability (DARTS) [387]. Si et al. developed a MALDI-TOF-based assay that can differentiate analogs of antibacterial natural products from mutated bacterial colonies directly from the MALDI target plate without the need for any liquid handling in a high-throughput fashion [404]. Gurard-Levin et al. developed a label-free HTS assay using self-assembled monolayers (SAMs) of alkanethiolates on gold with matrix-assisted laser desorption/ionization (MALDI) time-of-flight (TOF) MS, a technique named SAMDI, and screened 102,400 compounds in 24 h against lysine deacetylase 8 (KDAC8), formerly known as histone deacetylases or HDACs, which removes acetyl moieties from the N^ε^ amino group of lysine residues and plays a role in regulating gene expression, diabetes, and cancer [405]. This label-free approach does not require the use of any chromophore reagents, dyes, or antibodies; however, it only screens the substrate binding of a specific protein in a targeted manner. Mass cytometry enabled the classification of cell populations based on metabolite profile [406].

Other biophysical approaches that can detect the binding of a ligand to a target molecule include surface plasma resonance, thermal shift analysis, fluorescence polarization, scintillation proximity assay, Forster Resonance Energy Transfer (FRET), isothermal titration calorimetry, and various other biochemical assays that have been successfully used to identify ligand–enzyme interaction such as biotin carboxylase, DNA gyrase, cell division protein FTsZ, TetR family transcriptional regulatory repressor ethR, and β-lactamase, and are comprehensively discussed elsewhere [407,408,409]. Newer biophysical approaches to delineate target–ligand interaction such as in-cell NMR [410] and in-cell or cellular thermal shift assays (CETSAs) involve exposing a molecular target to a complex mixture in its native cellular environment followed by quantitative mass spectrometry-based target identification [411]. The readers are encouraged to consult the excellent reviews on the application of biophysical approaches in HTS and drug discovery to know details about these techniques and their suitability cited here [313,412,413]. Several groups are also working to screen libraries to identify molecules that could inhibit the production of colored compounds that act as a virulence factor, such as STX inhibitors against MRSA, pyocyanin inhibitors against *P. aeruginosa*, melanin inhibitors against *C. neoformans*, Hz inhibitors against *P. falciparum,* etc., and a comprehensive discussion on this can be found in the review by Ni et al. [414]. Other emerging technologies such as biolayer interferometry (BLI), microscale thermophoresis (MST), surface acoustic waves, second-harmonic generation, waveguide-based grating-coupled interferometry, and electrically switchable nanolevers that are based on photometry, fluorometry, wave, and temperature gradient may be explored in the future for antibacterial drug discovery research. Irrespective of the various innovative strategies for HTS assays, finding new antibacterial agents, as well as determining their mechanisms of action, have been very challenging, and da Cunha et al. reviewed various techniques such as overexpression and knockout-based genetics, promoter–receptor libraries, transcriptomics, proteomics, metabolomics, bacterial cytological profiling, and vibrational spectroscopy that are currently being used in mechanism-based antibacterial drug discovery [415] that the readers are encouraged to consult.

## 7. Final Remarks and Future Perspectives

**Figure 12:**
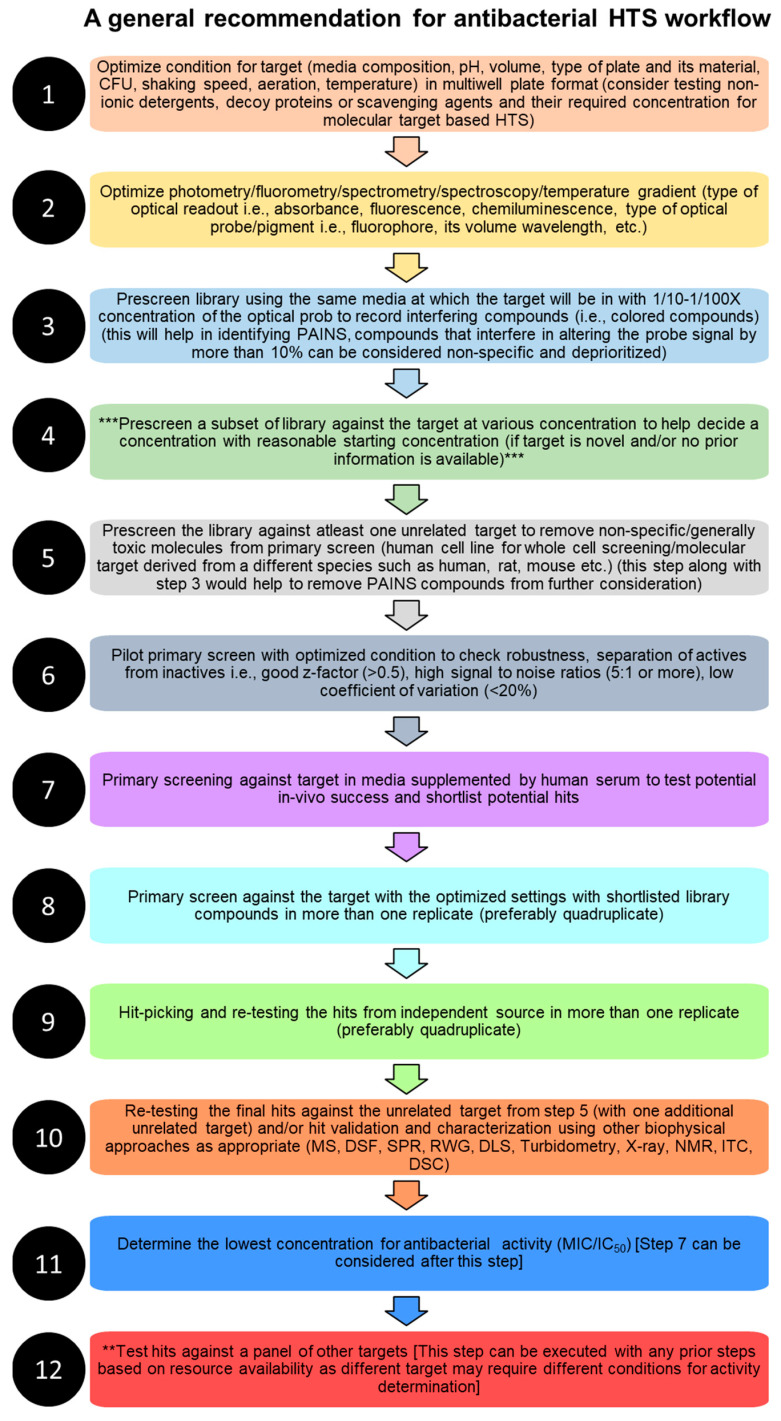
A general recommendation for HTS designing for antibacterial drug discovery. Steps 4 and 12 are starred to indicate they are optional and depend on the availability of resources. MS—mass spectrometry, DSF—differential scanning fluorometry, SPR—surface plasmon resonance, RWF—resonant waveguide grating, DLS—dynamic light scattering, ITC—isothermal titration calorimetry, X-ray—X-ray crystallography, NMR—nuclear magnetic resonance, DSC—differential scanning calorimetry.

Success in discovering new antibacterial agents using natural products or synthetic molecule library screening has remained quite limited despite the availability of sophisticated instrumentation and technologies and smarter strategies. In this review, various strategies of HTS campaigns are discussed for antibacterial drug discovery using numerous examples with new and contemporaneous designs along with a discussion of the technical aspects needed for planning and implementing screening ventures. A general guideline to aid in decision-making (Figure 12) and designing HTS assays is proposed, and various supporting, complementary, and alternative technologies for HTS are discussed.

In light of this discussion, it can be reasonably said that to be able to come up with a new member of an existing antibiotic class or a completely new antibiotic class can be similar to an effort of finding a needle in a haystack and might require screening millions of chemical molecules or natural product synthesizers. Additionally, the short-term course for infectious disease treatment makes it difficult for antibiotics to compete with other classes of therapeutics from an economic benefit point of view, resulting in declining enthusiasm for antibacterial research by the stakeholders. Antimicrobial resistance awareness campaigns also encourage minimal use of antibiotics. All these hurdles reduce the return on investment for antibacterial drug discovery and development and deter pharmaceutical companies from further investment in this sector. Academic labs and research organizations, alone or in collaboration with smaller biotech companies, have picked up some of these challenges to find alternative approaches that have been discussed in this review. In the future, government initiatives and legislation around antibacterial use and research necessities might be required. These innovative approaches did not necessarily result in new antibiotics classes yet, but they expanded knowledge about bacterial physiology, pathogenicity, pathogen–host interaction, the mechanism of antibiotic action, and resistance development, which would be critical for future antibacterial drug discovery expeditions. New antibiotics might come from screening synthetic molecule libraries in a novel way that will enable access to novel targets or new natural sources. Novel and smarter ways have been continuously explored, as can be seen in selective screening, multi-target assays, microbial co-culture, screening externally stimulated microbial culture, and antivirulence compound screening. Notable technologies such as iChip, compound activity mapping, BioMap, droplet microarrays, etc., have demonstrated promising results and hold the potential for future antibacterial drug discovery initiatives. Past studies have contributed tremendous knowledge to a better approach and design for future high-throughput screening assays for antibacterial drug discovery, and newer ways are continuously pursued, holding the hand of new antibiotics that might see the light.

## 8. Conclusions

Bacterial infection is a major health threat that needs major global attention. The lack of antibiotics in the pipeline and the continued emergence of resistance are aggravating this critical health concern. The synthetic molecule library and natural product library both have potential and screening efforts, and involving them means that they can benefit from each other. In addition to traditional cellular or molecular target-based HTS assays, newer approaches are being pursued that include mechanism-informed phenotypic screening, screening for quorum-sensing inhibitors, multi-target assays, microbial co-culture, screening externally stimulated microbial culture, and various combination strategies. To date, more than 50% of antibiotics available on the market are derived from natural products. Despite posing severe challenges in collecting, growing, extracting, and screening natural products, they are still preferred for drug discovery assays due to their diverse chemical nature, privileged structure, and spatial arrangement, in addition to the fact that only a fraction of the natural resources have been screened so far with the majority being untapped. Microfabricated chips in particular demonstrated ultra-high-throughput in identifying antibiotic sensitivity and identified an anti-MRSA agent, teixobactin, from an uncultured bacterium by screening extracts of 10,000 isolates. Among other notable platforms are Compound Activity Mapping and BioMap, which can help to predict the identity and the mode of action of bioactive molecules from complex natural product extract libraries. Synthetic molecule libraries, on the other hand, are easy to obtain and can be rapidly screened against a variety of targets, both cellular and molecular. Recent attempts at using biomimetic conditions such as human serum, artificial urine, iron-depleted media, human ascites, macrophage-mimicking media, etc., helped to find newer antibacterial agents that would otherwise be missed and antibacterial targets that are crucial for bacterial growth and survival. Drug repurposing initiatives through screening microsomal drug metabolites of synthetic molecule libraries in the absence and presence of other antibiotics yielded drugs and their metabolites with improved and synergistic antibacterial activity. Miniaturized approaches using synthetic molecule libraries, such as droplet microarray (DMA) technology, demonstrated ultra-sensitivity in detecting antibacterial activity on a nano-liter scale, whereas high-performance thin layer chromatography-based HTS demonstrated synthesis, screening, and the identification of antibacterial products from 60 organic reactions at the nano-mole level. High-throughput synthesis and screening of peptides of diverse sizes, sequences, and structures are also being explored for antibacterial drug discovery with some success.

The success of an HTS assay greatly depends on the efficiency and robustness of its performance. Careful design and optimization including the appropriately chosen library and technology platform, storage conditions, the target of the assay, orthogonal assays for hit validation, toxicity screening, dereplication strategy, data analysis, error management, etc., are all very critical and ultimately determine the output of HTS assays. Due to pressing needs for new antibacterial agents, numerous auxiliary ways such as selective screening, genetic manipulation, synthetic biology, multiomic analysis, virtual screening, combinatorial chemistry, fluidic and image-based technologies, phage display, antibody-based technology, metal nanoparticles, and other biophysical approaches are continuously being pursued as novel, complimentary, and/or alternative means for HTS antibacterial assays. It can be difficult to predict the success of these approaches, and there is not really one clear winner enabling the discovery of novel antibiotics. Perhaps it is also time to revisit the abandoned and/or neglected molecules from the past due to the narrow spectrum of activity, adverse side effects, the lack of bactericidal activity, or undesired pharmacokinetic parameters and take the help of medicinal chemistry to modify them as observed in case of linezolid, daptomycin, and fidaxomicin; or to screen for agents that interfere with resistance, stimulate host immunity, and reduce virulence.

## Figures and Tables

**Table 1:** Major bottlenecks in natural product drug discovery [48,68,69,70].

(1) A lack of chemical and/or biological diversity in existing natural sources. (2) Challenges in procuring new sources. (3) Possible loss of sources. (4) Seasonal or environmental variations. (5) The preparation of an extract library for HTS assays. (6) Low abundance of bioactive entity for HTS detection. (7) Variable extraction approaches for various natural products. (8) Challenges in isolation, purification, and structure elucidation of the active component from the crude extracts. (9) Synergistic activity from multiple compounds that disappear upon purification. (10) The time it takes to perform all these in a high-throughput manner. (11) The risk of rediscovering previously identified bioactive molecules. (12) Interferences from compounds in the mixture that emit or absorb radiation or scatter light. (13) The presence of nuisance compounds such as phorbol esters, fatty acids, acidic polysaccharides, and polyphenolics such as tannins, flavonoids, and metal impurities. (14) The presence of toxic compounds, etc.

**Table 2:** Overview of various automated systems for high-throughput screening of natural products. (Adapted from Rienzo et al. [80] and reprinted with permission.)

	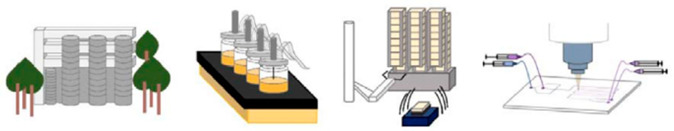			
	Manufacturing and Pilot Scale Bioreactor	Lab-Scale Bioreactor	Microplate Culture Model	Microfluidic Culture Model
Volume	100–200,000 L	0.25–30 L	0.1–1.0 mL	<100 nL
Throughput (genotypes/year)	<10	~10^3^	~10^6^	~10^9^
Cost per fermentation	USD 10 K–USD 200 K	~USD 1 K	<USD 0.5	<USD 0.0001
Advantages	Scalable, cost-effective	High-confident strain Manufacturing with high control	High parallelization to rank genotypes for further testing	Highest throughput with the smallest cost
Limitations	Hardware constraints hinder process control	Non-homogenous chemical environment	High false positives, irreproducibility, or unreliable readouts	Highly sophisticated instruments are required, very low sample size requires high sensitivity
Predictive power for commercial scale	–	Reliable	Variable	Unexplored

**Table 3:** Example of NPL-HTS for antibacterial drug discovery (this is not an exhaustive list).

Successful Hit	Target Organism	Protein Target	MIC ^a^/IC_50_^b^	Reference
Bischloroanthrabenzoxocinone (BABX) (12)	*E. coli* (MB4902) *S. aureus* (MB2985)	Fatty acid synthesis Type II (FASII)	0.2–0.4 μg/mL^a^	[120]
Anziaic acid (13)	*B. subtilis* (ATCC 6633) *E. coli* (BAS3023)	Topoisomerase I	6 μg/mL ^a^ 12 μg/mL ^a^	[121]
04E04 03F11 04E04 02F09, 02H08	*E. faecalis* *H. influenzae* *M. catarrhalis* *P. aeruginosa hypersensitive* (ATCC 35151)	Phenylalanyl-tRNA Synthetases (PheRS)	8 μg/mL ^a^ 4 μg/mL ^a^ 1 μg/mL ^a^ 4 μg/mL ^a^	[122]
Resveratrol Tetramer (-)-Hopeaphenol (14)	*Y. pseudotuberculosis*	Type III secretion	6.6 μM ^b^	[123]
NAT13-338148 NAT18-355531 NAT18-355768	*Clostridioides difficile* (ATCC BAA 1870)	ND	0.5–2 μg/mL ^a^	[124]
3-methoxynordomesticine (15)	*Mycobacterium tuberculosis* H37Rv *Mycobacterium bovis* BCG	MurE (IC50 < 100 µM)	≤5 μg/mL ^a^	[125]

^a^ MIC—Minimum Inhibitory Concentration. ^b^ IC_50_—Concentration at which a substance exerts half of its maximal inhibitory effect. ND—not determined.

**Table 4:** Example of NPL-HTS assays for antibacterial drug discovery (this is not an exhaustive list).

HTS Strategy			Library Size and Name	Z/Z′-Score	Target	Hits (ID/name)	Activity *	Cytotoxicity Tested	Reference
MT	CT	C							
✔		✔	20,000 (ChemBridge)	0.82	N-acetylglucosamine-1-phosphate uridyltransferase (GlmU)(acetyltransferase domain)	6624116 ^a^ 5655606 ^a^ 5810599 ^a^ 6012954 ^a^	65.15 ± 3.31 µM ^b^ 18.58 ± 0.81 µM ^b^ 9.01 ± 0.04 µM ^b^ 38.84 ± 0.29 µM ^b^	Human liver hepatocellular carcinoma cells (HepG2 cells)	[190]
					*M. tuberculosis* H37Rv	6624116 ^a^ 5655606 ^a^ 5810599 ^a^ 6012954 ^a^	16 µg/mL ^c^ 2 µg/mL ^c^ >32 µg/mL ^c^ >32 µg/mL ^c^		
✔			50,000 (Maybridge)	0.71	N-acetylglucosamine-1-phosphate uridyltransferase (GlmU) (acetyltransferase domain)	MAC0021939 MAC0008028 MAC0029665	189 ± 17.7 nM ^d^ 1.11 ± 0.08 ×10^3^ nM ^d^ 28.2 ± 1.83 nM ^d^	Not tested	[191]
	✔		20,502 (Broad Institute)	0.7–0.8	*M. tuberculosis* H37Rv, *M. bovis* BCG strain Pasteur, and *M. smegmatis* MC2155	Benzimidazole derivative ^e^ Nitro-triazole derivative ^f^	37.5 µM ^c^ 0.488 µM ^g^	Not tested	[192]
					Membrane protein large 3 (MmpL3) (target for ‘e’)Decaprenylphosphoryl-β-d-ribose 2′-epimerase (DprE1) (target for ‘f’)				
	✔		57,000 (Timtec, Cerep, and ChemBridge)	<0.5	*Mycobacterium tuberculosis* H37Rv, H37Ra and BCG Pasteur	DNB1 ^h^ DNB2 ^i^	0.2 µM ^c^ 0.2 µM ^c^	Mouse bone marrow-derived macrophages	[193]
					Decaprenylphosphoryl-β-D-ribose 2′-epimerase (DprE1/DprE2)				
	✔		125,000	Not reported	ΔtolC *Escherichia coli*	Eight compounds containing 2-pyrazol-1-yl-thiazole scaffold	0.037–8 µg/mL ^c^	HEK293 cells A549 cells MCF7 cells	[194]
	✔		17,500 (Chemical Biology Consortium Sweden Primary Screening Set Collection)	Not reported	*Streptococcus pneumonia* T4	THCz-1 (1-amino substituted Tetrahydrocarbazole)	1.3 µg/mL^c^	Lung epithelial A549 cells	[195]
					Undecaprenyl pyrophosphate-containing lipid intermediates				
	✔		28,300 LOPAC: 1408 compounds Echaz microcollection: 7304 compounds CDI collection: 17,000 NCH collection: 154 secondary myxobacteria metabolites Library, various sources: 1936 synthetic organic molecules Peptide library: 1045 short linear or cyclic peptide	0.5–0.9	*Vibrio cholera* N16961 and NM06-058	vz0825 vz0500 1541-0004	1.6 µM ^c^ 3.1 µM ^c^ 6.3 µM ^c^	Mouse fibroblast cell line L929	[189]
					K^+^-channel sensor histidine kinase (KdpD)				
	✔		20,338 LOPAC: 1408 compounds Echaz microcollection: 7304 compounds CDI collection: 17,000	0.5–0.9	0139 *V. cholerae* MO10 (aphA transcript)	vz0761 vz0852 53760866	10 µM ^c^ 6 µM ^c^ 38 µM ^c^	Mouse fibroblast cell line L929 ^j^	[196]

MT-HTS—molecular target-based HTS, CT-HTS—cellular target-based HTS, C-HTS—computational HTS. ^a^ ChemBridge ID. ^b^ IC_50._^c^ MIC. ^d^ K_iu_—uncompetitive inhibition constant. * Activity is listed based on safety for mammalian cells if tested, otherwise it is based on reported potency. ^e^ N-(2,4-dichlorobenzyl)-1-propyl-1H-benzo[d]midazole-5-amine. ^f^ 1-(4-(tert-butyl)benzyl)-3-nitro-1H-1,2,4-triazole. ^g^ IC90. ^h^ Di-nitro-methoxy benzamide. ^i^ Di-nitro-fluoro benzamide. HEK293—human embryonic kidney 293. A549—adenocarcinomic human alveolar basal epithelial. MCF7—Michigan cancer foundation-7. ^j^ In vivo study performed; 80% reduction in colonization in mice with 53760866. Blue text indicates Z-score. Magenta text indicates Z’-score. The orange shaded region indicates the targets (cellular bacteria, molecular protein) under study. The green shaded region indicates the molecular target identified by the mechanism of action query experiment post-cellular or computational HTS.

**Table 5:** Synergy among different antibacterial agents against various bacteria, including multidrug and extensively drug-resistant strains from HTS assays (this is not an exhaustive list).

Target Bacteria	Compounds Showing Synergy	Reference
*Acinetobacter baumannii* strain AB5075	5-fluorouracil and azithromycin Colistin sulfate with fluspirilene and Bay 11-7082	[218]
MRSA (ATCC 43300)	Cefoxitin with floxuridine, gemcitabine, novobiocin, rifaximin, 4-quinazolinediamine, celastrol, and teniposide	[220,222,227]
	Ceftobiprole with cloxacillin, cefotaxime, oxacilline, flucloxacillin, dicloxacillin, nafcillin, imipenem, meropenem, cefoxitin, piperacillin, and tazobactam	[228]
	Ceftaroline with cloxacillin	
MRSA USA300	Cefuroxime and ticlopidine	[229]
MRSA N315	Meropenem, piperacillin, and tazobactam	[230]
MRSA ATCC 29213 *Pseudomonas aeruginosa*	IITR00693 (2-Aminoperimidine) and polymyxin B	[231]
*Pseudomonas aeruginosa* lasB-gfp (ASV)	Auranofin and colistin	[232]
*P. aeruginosa* (Clinical isolates)	Tetracycline and polyamine scaffolds containing molecules	[233]
*P. aeruginosa* ATCC 27853	Amikacin and ceftriaxone	[234]
*P. aeruginosa* P6540	Rifampicin and imipenem Colistin and imipenem	[235]
*M. smegmatis* mc^2^155 *M. tuberculosis* H37Rv (ATCC 27294)	Spectinomycin and bromperidol	[236]
*M. tuberculosis* H37Rv (ATCC 27294)	Rifampicin with Compound 5655606	[190]
*Legionella pneumophila* serogroup 1 (Lp02::flaA::lux)	Rifampin, azithromycin, and minocycline in any combination	[237]
Carbapenem-resistant enterobacteriaceae	Triclosan and meropenem	[238]
Trimethoprim-resistant clinical *E. coli*	Azidothymidine with trimethoprim and sulfamethizole	[239]
*Pandoraea nosoerga* P8103	Rifampicin and minocycline	[235]
*Burkholderia multivorans* P6539	Fluoroquinolones and β-lactams	
*E. coli* BW25113 *P. aeruginosa* PA01	Novobiocin with pivmecillinam and echinomycin Novobiocin with niridazole	[240]
*Klebsiella pneumoniae* N11-2218	Meropenem with aspergillomarasmine A	[241]
